# Interpretable Recalibration of a Sequential Organ Failure Assessment–Derived Score Using Machine Learning–Guided Optimization: Multicenter Cohort Study

**DOI:** 10.2196/99361

**Published:** 2026-11-13

**Authors:** Juan Dueñas-Ruiz, Yanelis Manzano Nieves, Anny María Hernández Cuello, Jaime Azpiazu Saiz, Ancor Sanz-García, Agustín Mayo-Íscar, Francisco Martín-Rodríguez, Isabel de la Torre

**Affiliations:** 1 Faculty of Medicine, Universidad de Valladolid, Avda. Ramón y Cajal, 7, Valladolid, 47005, Spain, +34 983 42 30 00; 2 Artificial Intelligence Department, CIDAUT Foundation, Valladolid, Spain; 3 European University of the Atlantic, Santander, Spain; 4 International Iberoamerican University, Campeche, Mexico; 5 International University of Cuanza, Cuito, Angola; 6 International University Foundation of Colombia, Bogotá, Colombia; 7 University of La Romana, La Romana, Dominican Republic; 8 Faculty of Health Sciences, Universidad de Castilla la Mancha, Talavera de la Reina, Spain; 9 Technological Innovation Applied to Health Research Group (ITAS Group), Faculty of Health Sciences, University of Castilla-La Mancha, Talavera de la Reina, Spain; 10 Health Care Evaluation Research Group (ECUSAL), Instituto de Investigación Sanitaria de Castilla-La Mancha (IDISCAM), Toledo, Spain; 11 Department of Statistics and Operative Research, Faculty of Medicine, University of Valladolid, Valladolid, Spain; 12 Valladolid Health Research Institute (IBioVALL), Valladolid, Spain; 13 Instituto de Investigación en Matemáticas (IMUVA), Universidad de Valladolid, Valladolid, Spain; 14 Emergency Medical Services (SACYL), Valladolid, Spain; 15 Department of Signal Theory and Communications, University of Valladolid, Valladolid, Spain

**Keywords:** organ dysfunction scores, machine learning, critical care, hospital mortality, intensive care units, interpretability

## Abstract

**Background:**

The Sequential Organ Failure Assessment (SOFA) score is central to risk stratification in intensive care units (ICUs), but its thresholds and 0- to 4-point increments were set by expert consensus in 1996 and never reestimated from outcome data. Machine learning models discriminate better while discarding the additive, point-based structure that makes SOFA auditable at the bedside, and methods for updating a legacy score rather than replacing it remain scarce.

**Objective:**

This study aimed to develop and internally validate a data-driven recalibration of a SOFA-derived score that learns thresholds and integer weights from outcome data while preserving an additive, interpretable point-based structure and to test whether it improves discrimination for ICU mortality over a prespecified SOFA-derived reference score.

**Methods:**

We conducted a retrospective multicenter cohort study using the eICU Collaborative Research Database (208 US hospitals, 2014‐2015). We analyzed 14,848 adult first ICU admissions with complete day 1 SOFA-related measurements; 133,684 (90%) of 148,532 encounters with outcome data were excluded because of at least 1 missing determination. As vasoactive dose units could not be harmonized and urine output was largely unavailable, a prespecified SOFA-derived reference score served as the comparator. The framework couples supervised discretization (univariate decision trees and interval number chosen by a penalized validation criterion) with constrained integer-weight optimization under monotonicity, integrality, and scale-anchoring constraints, plus logistic calibration. Performance was estimated from pooled out-of-fold predictions of 10-fold nested cross-validation grouped by hospital, with bootstrap CIs; final thresholds and weights were refit on the full cohort and contributed no performance estimate.

**Results:**

ICU mortality was 17.03% (2529/14,848). The recalibrated score achieved an area under the receiver operating characteristic curve (AUROC) of 0.811 (95% CI 0.800‐0.819) versus 0.751 (95% CI 0.740‐0.761) for the reference score (paired difference 0.059, 95% CI 0.053‐0.067), and an area under the precision-recall curve of 0.495 (95% CI 0.477‐0.515) versus 0.393 (95% CI 0.377‐0.411; paired difference 0.102, 95% CI 0.087‐0.116). Improvement was consistent across all 10 hospital-grouped folds and length-of-stay strata. After unweighted logistic recalibration, global calibration was adequate; the Brier score was lower (0.112 vs 0.123), whereas other calibration-error measures were higher. Discrimination approached unconstrained XGBoost (Extreme Gradient Boosting; AUROC 0.818) and exceeded penalized logistic regression (AUROC 0.775) fitted on the same folds. The advantage persisted in all 1000 resampling replications and in a prespecified sensitivity analysis expanding the cohort to 61,469 encounters (median paired AUROC difference 0.037).

**Conclusions:**

Data-driven recalibration under explicit structural constraints can update the cutpoints and integer weights of an established clinical score without abandoning its additive, bedside-computable form and should transfer to other legacy instruments. The recalibrated score is a mortality risk model on its own 0‐71 scale; an open-source calculator is provided, and external and prospective validation is required before clinical use.

## Introduction

### Background

Accurate and timely assessment of organ dysfunction remains fundamental to mortality prediction and clinical decision-making in the intensive care unit (ICU). The Sequential Organ Failure Assessment (SOFA) score represents a cornerstone tool in critical care medicine, providing clinicians with a quantitative framework to assess organ dysfunction and predict short-term mortality [1]. Originally developed in 1996 [2] (Table S1 in Multimedia Appendix 1) and updated in 2025 [3] (Table S2 in Multimedia Appendix 1), the SOFA score evaluates 6 organ systems—respiratory, cardiovascular, hepatic, coagulation, renal, and neurological—assigning points based on objective clinical and laboratory parameters. This scoring system has demonstrated remarkable utility in stratifying patient risk and informing clinical decision-making across diverse critical care settings [4].

Beyond its predictive value for mortality, the SOFA score serves as a dynamic monitoring tool that captures the trajectory of organ dysfunction over time. The score has been validated in numerous patient populations, including patients with sepsis, acute respiratory distress syndrome, or trauma and patients after cardiac arrest, demonstrating its versatility across heterogeneous ICU cohorts [5]. Furthermore, the SOFA score has been incorporated into sepsis definitions (Sepsis-3 criteria) and quality improvement initiatives, solidifying its role as an essential metric in critical care practice [6].

Despite its clinical utility, the SOFA score was originally developed through expert consensus, with physiological thresholds and point increments defined heuristically rather than derived from outcome-driven data [2]. Each organ domain is discretized into fixed intervals scored from 0 to 4, implicitly assuming equal contributions across score increments and stability of threshold relevance over time. These assumptions may no longer hold in contemporary ICU populations.

Changes in case mix, baseline comorbidity burden, and advances in organ support therapies have altered both the distribution and prognostic impact of key physiological variables [7,8]. Several studies have highlighted limitations of SOFA in modern settings, including imperfect calibration, reduced discrimination in some subgroups, and challenges in accurately capturing neurological dysfunction and chronic organ impairment [5,9,10]. Moreover, SOFA does not distinguish acute organ failure from chronic baseline dysfunction, potentially conflating prognostic signals in patients with preexisting disease [9].

In response to these limitations, there have been calls to revise or update SOFA using contemporary data. Recent large-scale analyses combining expert input with outcome-based modeling have demonstrated that data-driven recalibration of organ failure thresholds is feasible and clinically meaningful [3]. However, such updates remain infrequent, and the core additive structure of SOFA has remained largely unchanged for decades.

Recent advances in AI and machine learning (ML) have opened new avenues for enhancing traditional clinical scoring systems. Multiple studies have demonstrated that AI algorithms can identify complex, nonlinear relationships within clinical data that conventional linear models may overlook [11,12]. ML models have demonstrated strong predictive performance for clinically relevant outcomes in critical care [13]. These AI-enhanced approaches leverage large datasets to recalibrate the weighting of individual organ dysfunction components, potentially capturing temporal patterns and patient-specific trajectories that static scoring systems cannot [14].

However, many high-performing ML models function as black boxes, relying on complex feature interactions that are difficult to reconcile with clinical reasoning. This lack of transparency has been identified as a major barrier to clinical trust and real-world adoption in high-stakes medical settings [15]. Although post hoc explanation techniques such as Shapley Additive Explanations [16] or Local Interpretable Model-Agnostic Explanations [17] are commonly used to interrogate black box models, they operate outside the model’s core logic and may provide unstable or clinically unintuitive explanations [18]. In contrast, additive point-based models remain preferred by many clinicians because they are transparent, verifiable, and aligned with established clinical intuition. This trade-off between accuracy and interpretability has motivated growing interest in hybrid modeling strategies that integrate ML with clinically meaningful structures.

### Related Work and Gap in the Literature

Recent advances in interpretable ML for health care have led to the development of data-driven frameworks for constructing transparent clinical risk scores that combine statistical learning with clinically meaningful representations. Score generation frameworks such as AutoScore [19] have demonstrated that data can produce new additive integer scores with performance comparable to regression approaches. These methods address the problem of constructing scores de novo. This work addresses a complementary problem: updating the parameters of an existing, widely adopted score without altering its structure.

Complementary approaches have further expanded the space of interpretable scoring models. FasterRisk, for instance, formulates score construction as a constrained optimization problem and uses efficient beam search strategies with rounding to produce a set of near-optimal sparse scoring systems [20]. Other methods, such as SymScore, use symbolic regression with monotonicity constraints to optimally group responses and assign integer weights, yielding interpretable additive score tables; it has been applied to shorten existing clinical questionnaires while approximating their total score [21]. Collectively, these approaches demonstrate a growing methodological toolkit for deriving interpretable, data-driven risk scores that align with clinicians’ preference for simple additive models.

In parallel, hybrid modeling strategies have been proposed to reconcile predictive performance with interpretability by explicitly incorporating clinical structure into ML architectures. One recent example is a tree-structured hybrid model for ICU mortality prediction, in which patient features are grouped into clinically motivated modules corresponding to different organ systems or disease processes. Each module is learned from data, while the overall structure reflects established pathophysiological knowledge of multiorgan dysfunction. The outputs of these modules are subsequently aggregated to generate a final risk estimate, allowing interpretation at the level of organ systems or clinical domains. While such hybrid frameworks improve transparency relative to black box models, they typically rely on multiple interacting submodels and do not yield a single, simple scoring rule that can be readily compared to existing clinical scores [22].

Despite these advances, relatively few efforts have focused on recalibrating established clinical scoring systems that are already deeply embedded in routine practice. Widely used severity and risk scores, such as Acute Physiology and Chronic Health Evaluation (APACHE), Simplified Acute Physiology Score (SAPS), and SOFA, were largely developed decades ago using expert consensus and limited datasets. Although numerous ML models have since demonstrated superior predictive performance, these legacy scores remain in widespread use due to their familiarity, interpretability, and extensive validation across clinical settings.

Given the need to modernize aging scoring systems, large-scale collaborative initiatives have occasionally undertaken comprehensive revisions. A notable example is the recent development of SOFA 2.0, an updated organ dysfunction score informed by contemporary ICU practice and large international datasets [1]. This effort combined expert consensus methods with extensive empirical analysis of more than 1 million ICU admissions to revise variable definitions, introduce additional measurements, and adjust thresholds across organ systems. Importantly, SOFA 2.0 preserved the original additive structure and the familiar 0 to 4 per-organ scale, thereby maintaining continuity with the original score while improving its relevance to modern critical care. However, the development of SOFA 2.0 required substantial expert involvement, bespoke data curation, and a resource-intensive consensus process, limiting its generalizability as a methodological template.

More broadly, there remains a clear methodological gap in the literature: the absence of widely adopted, general, automated approaches for outcome-driven recalibration of legacy clinical scores that preserve an additive structure grounded in the clinical domains of the original score. Traditional statistical recalibration techniques typically focus on adjusting model intercepts or recalibrating predicted probabilities in new populations, without modifying the internal composition of the score itself. While such approaches can improve calibration, they do not offer a principled way to update variable thresholds or point allocations in longstanding scoring systems as clinical practice and patient populations evolve [23]. Consequently, when established scores exhibit signs of performance degradation or miscalibration, clinicians are often faced with a binary choice between continued use of an outdated tool or adoption of an entirely new model that sacrifices interpretability and historical continuity. Even in the case of SOFA 2.0, maintaining comparability with the original score has required explicit mapping strategies and periods of dual scoring, underscoring the challenges of updating entrenched clinical instruments.

The widespread deployment of electronic health records (EHRs) in intensive care settings creates an opportunity to leverage routinely collected clinical data for continuous model refinement. Large-scale, publicly available critical care databases—such as Medical Information Mart for Intensive Care IV (MIMIC-IV) and eICU—now provide multicenter data spanning diverse patient populations and institutional practices, enabling robust development and external validation of recalibrated scoring systems. Unlike static scoring systems, data-driven recalibration frameworks can be systematically updated as patient populations evolve, supporting periodic parameter updating of decision support tools that maintain clinical validity over time. Crucially, preserving the interpretable, additive structure of established scores facilitates integration into existing EHR-based workflows and supports clinician trust in automated risk estimates.

To our knowledge, few existing frameworks provide automated, outcome-driven updating of a widely used legacy-score structure while retaining an interpretable, additive, point-based format. Addressing this gap would enable systematic, data-driven modernization of established clinical scores without abandoning the additive structures and clinical familiarity that underpin their sustained adoption.

### This Work

In this study, we address this gap by developing a data-driven recalibration of a SOFA-derived score that preserves an additive, point-based structure and full clinical interpretability. We introduce a modeling framework that learns optimal threshold cutpoints and interval-specific weights for each SOFA component directly from outcome data. By imposing explicit structural constraints, the approach ensures that each organ system contributes through transparent, clinically interpretable intervals rather than opaque transformations.

By retaining the organ-domain organization and additive, point-based logic of SOFA, the recalibrated score preserves clinical familiarity while allowing thresholds and weights to be updated from data. Importantly, the recalibrated SOFA-derived score is not proposed as a replacement for the canonical SOFA assessment: it addresses mortality risk estimation while preserving the additive structure of SOFA, but its total is not expressed on the conventional 0 to 24 SOFA scale. Through this work, we aim to demonstrate a practical pathway toward interpretable ML in critical care, one that balances predictive performance, robustness, and clinical credibility.

The main contributions of this study include (1) the development of a constrained optimization framework that updates a SOFA-derived score for contemporary ICU populations while preserving its additive structure and clinical transparency; (2) the incorporation of data-driven discretization and integer-weight optimization techniques that maintain full interpretability of the scoring system; and (3) internal validation on a large multicenter ICU dataset (208 US hospitals), demonstrating enhanced discrimination relative to the SOFA-derived reference score. Table 1 summarizes how this study differs from existing approaches. The aim of this study was to develop and internally validate a data-driven recalibration of a SOFA-derived score that preserves its additive, point-based structure and to test the hypothesis that outcome-driven optimization of its cutpoints and integer weights improves discrimination for ICU mortality relative to a prespecified SOFA-derived reference score, without loss of interpretability.

**Table 1.** Synthesis of related work and positioning of this study.

Approach	Uses exact legacy variable set?	Output format	Recalibrates a legacy score?	Preserves additive structure and comparability with original score?	Fully automated (no expert consensus)?
AutoScore [19]	No	New additive integer score	No	No	Yes
FasterRisk [20]	No	Sparse additive scoring system	No	No	Yes
SymScore [21]	No	Additive score table for shortened questionnaires	No	No	Yes
Tree-structured hybrid ICU^a^ mortality model [22]	No	Multimodule risk model with organ-level interpretation	No	No	Yes
SOFA^b^ 2.0 [1]	Partial	Updated SOFA with 0 to 4 per-organ additive scale	Yes	Partial—requires explicit mapping and dual scoring	No (by design—expert consensus approach)
Traditional statistical recalibration [23]	Yes	Same score with adjusted output calibration	Predicted risk only; thresholds, points, and total unchanged	Yes (no change to thresholds or weights)	Yes
This work	Partial	Recalibrated SOFA-derived additive point-based score (0‐71 scale)	Yes	Partial—additive structure preserved; no direct score equivalence.	Yes

a ICU: intensive care unit.

b SOFA: Sequential Organ Failure Assessment.

## Methods

### Data Source

This retrospective multicenter cohort study used the eICU Collaborative Research Database (eICU-CRD), a publicly available repository of deidentified EHR data from adult ICUs across the United States. The database contains 200,859 ICU stays for 139,367 unique patients admitted between 2014 and 2015 to 335 units at 208 hospitals participating in the Philips eICU telehealth program. Available data include high-resolution bedside monitor vital signs, nurse-validated observations, laboratory measurements, medication and infusion records, procedures, APACHE IV/IVa severity of illness scores and diagnostic codes, care plan documentation, and detailed administrative and outcome information, all stored as time-stamped structured data [24].

The eICU-CRD was selected for its large sample size, multicenter design, and high temporal granularity of physiological and treatment variables, making it well suited for developing and validating ML models in critical care. The database is freely accessible to qualified investigators via PhysioNet after completion of required ethics training and acceptance of a data use agreement.

### Study Population

We included adult patients (aged ≥18 y) with their first ICU admission in eICU-CRD for whom all SOFA-related physiological and laboratory measurements were available during the first 24 hours of ICU admission. After applying inclusion criteria and the preprocessing described in the following section, 14,848 patients were included in the final analysis. The detailed selection process is presented in Figure 1.

**Figure 1.**
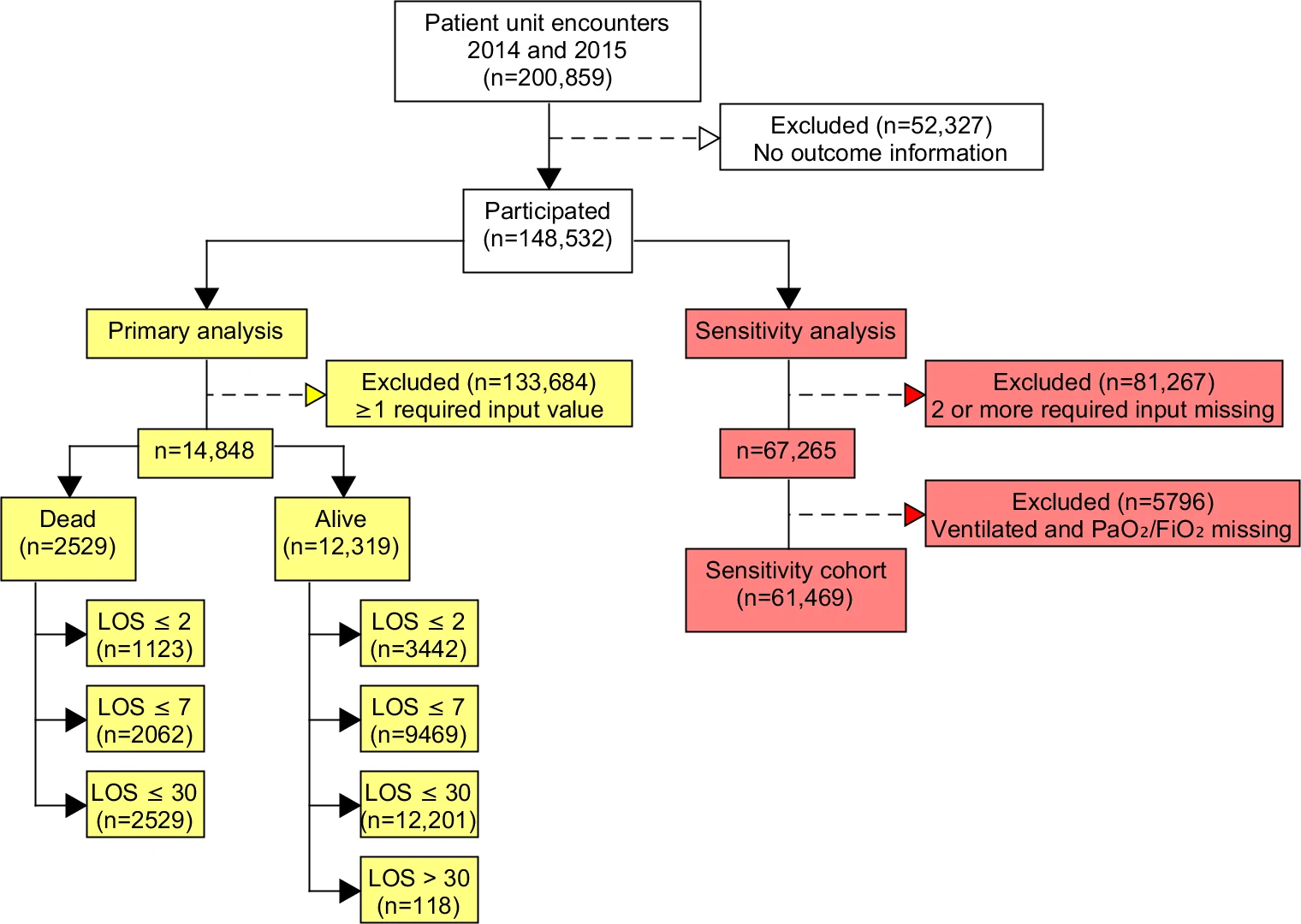
Flow diagram of patient inclusion for the primary and sensitivity analyses. Patient unit encounters from 2014 to 2015 were screened, and the resulting cohorts were defined according to data availability. The primary cohort was further stratified by intensive care unit mortality and length of stay (LOS). FiO₂: fraction of inspired oxygen; PaO₂: arterial partial pressure of oxygen.

### Data Extraction

All SOFA-related variables were obtained from 3 eICU-CRD tables: apacheApsVar, infusionDrug, and lab. From apacheApsVar, we extracted variables directly corresponding to SOFA components: mechanical ventilation status, Glasgow Coma Scale (GCS) total score, and mean arterial pressure (MAP).

Continuous infusions of the 4 SOFA vasoactive agents were extracted from infusionDrug. As dosing units could not be reliably harmonized across participating hospitals, each agent was encoded as a binary indicator of administration during the first ICU day.

Laboratory measurements (serum creatinine, total bilirubin, partial pressure of arterial oxygen [PaO₂], fraction of inspired oxygen [FiO₂], and platelet count) were obtained from the lab table, restricting records to samples drawn within the first 24 hours after ICU admission. For each patient and analyte, the most extreme (worst) value in that window was retained, following the convention used in APACHE for day 1 physiology. Age, sex, and ICU length of stay were additionally extracted from demographic tables to characterize the study population. The patient-level SOFA dataset was constructed by merging these derived variables using the unique patientUnitStayId identifier.

For the primary analysis, encounters were retained only when all continuous determinations required for the common analytic input set were available: paired PaO_₂_ and FiO_₂_ values required to calculate PaO_₂_/FiO_₂_, MAP, bilirubin, creatinine, and platelet count. Missing GCS components were handled according to a prespecified operational rule rather than used as an exclusion criterion: encounters receiving mechanical ventilation were assigned GCS a score of 7, whereas nonventilated encounters were assigned a score of 15. No other imputation was performed in the primary analysis.

As vasoactive-agent dose units could not be reliably harmonized across participating hospitals and urine output was incompletely available, the canonical original SOFA score could not be reconstructed exactly. We therefore prespecified a SOFA-derived reference score. Respiratory, neurological, coagulation, hepatic, and creatinine-based renal components followed the original SOFA thresholds for PaO₂/FiO₂ (mm Hg, with mechanical ventilation incorporated in the respiratory criteria), GCS, platelet count (×10⁹/L), total bilirubin (mg/dL), and serum creatinine (mg/dL), respectively. Urine output was not used for renal scoring. The cardiovascular component used MAP (mm Hg) and binary indicators of vasoactive-agent administration: MAP >70 mm Hg was assigned 0 points; for MAP ≤70 mm Hg, absence of all 4 vasoactive agents was assigned 1 point; dopamine, dobutamine, or epinephrine without norepinephrine was assigned 2 points; norepinephrine alone was assigned 3 points; and norepinephrine together with any of dopamine, dobutamine, or epinephrine was assigned 4 points. Laboratory variables used the most extreme value recorded within the first 24 hours, physiological variables obtained from apacheApsVar used the day 1 value selected according to the APACHE extraction logic described earlier, and vasoactive exposure denoted any administration during the first ICU day. Missing GCS components and the complete-case requirements were handled according to the prespecified rules described earlier. This SOFA-derived score served as the reference comparator in all paired performance analyses.

All data extraction and preprocessing were implemented in Python (version 3.10; Python Software Foundation) using pandas (The pandas development team), NumPy (NumPy Developers), and pyarrow (Apache Software Foundation).

### Model Development: Discretized Additive Risk Score

To construct an interpretable mortality prediction model, all continuous predictors were transformed into data-driven categorical intervals. The framework proceeds in 2 sequential stages: supervised discretization of each variable, followed by constrained optimization of integer interval weights.

#### Stage 1: Supervised Discretization

For each variable $x_{j}$, a univariate decision tree was fitted on the training set to identify potential split points ($k_{j}$ intervals). Each univariate decision tree was fitted using the Gini impurity criterion, with the binary ICU mortality label as the target. Tree depth was implicitly controlled by the candidate number of splits $k\in [2,K_{max}]$, where $K_{max}\text{=}5$ was selected as a practical upper bound that balances granularity against the risk of overfitting.

For each candidate discretization, bin-level mortality probabilities were estimated on the training data using Laplace-smoothed empirical risks. These probabilities were then applied to the validation set to compute the corresponding log loss. To avoid overly granular discretizations, a linear complexity penalty proportional to the number of intervals was added. The value of $k_{j}$ minimizing the penalized validation loss was selected. When the decision tree did not yield the required number of split points, the remaining thresholds were completed using empirical quantiles, ensuring a consistent number of intervals across variables. The resulting thresholds $\left\{t_{j,b}\right\}$, where $t_{j,0}\text{=}\text{-}\infty$ and $t_{j,k_{j}}\text{=}\infty$, defined ordered, nonoverlapping intervals $\left(t_{j,b},t_{j,b\text{+}1}\right]$, yielding data-driven ranges derived from outcome data rather than predefined cutoffs.

Each interval was encoded using a binary indicator function


$$I_{j,b}\left(x_{j}^{\left(i\right)}\right)=\left\{ \begin{array}{ll} 1, & \text{if }t_{j,b}§lt;x_{j}^{\left(i\right)}\leq t_{j,b+1},\\ 0, & \text{otherwise}. \end{array}\right.$$

where $x_{j}^{\left(i\right)}$ denotes the value of variable j for patient i. For each variable, exactly 1 indicator is active per patient, enabling a transparent mapping from raw clinical values to interval-based features.

#### Stage 2: Constrained Integer Weight Optimization

##### Overview

An additive scoring function aggregated contributions across all variables:


$$s(x^{\left(i\right)})\text{=}\sum_{j\text{=}1}^{d}\sum_{b\text{=}0}^{k_{j}\text{-}1}w_{j,b}I_{j,b}\left(x_{j}^{\left(i\right)}\right)$$

where each weight $w_{j,b}$ represents the effect associated with belonging to a specific clinical range. This structure mirrors traditional clinical risk scores such as SOFA or APACHE, while allowing sequential data-driven estimation of interval boundaries and optimization of their associated contributions.

Weights were constrained to be integers to enhance interpretability and facilitate bedside implementation and were bounded to -W,W for numerical stability.

##### Monotonicity Constraints

Each domain of the original SOFA score is monotonic by construction: points increase as the PaO_₂_/FiO_₂_ ratio decreases, the GCS score decreases, the platelet count decreases, creatinine and bilirubin levels increase, respiratory and vasoactive support escalate, and respiratory support is required. As this framework claims to preserve that structure, monotonicity was imposed as a constraint rather than left to the data. Let $J^{\text{+}}$ denote variables for which risk is nondecreasing with increasing raw values and $J^{\text{-}}$ those whose risk is nonincreasing:


$$w_{j,0}\leq w_{j,1}\leq \cdots \leq w_{j,k_{j}\text{-}1},j\in J^{\text{+}}$$

$$w_{j,0}\geq w_{j,1}\geq \cdots \geq w_{j,k_{j}\text{-}1},j\in J^{\text{-}}$$

Binary indicators, including vasoactive agent use and respiratory support, were treated as 2-level ordered variables. For these variables, the monotonicity constraint reduces to a sign restriction requiring the contribution associated with exposure or intervention to be no lower than that associated with nonexposure.

##### Exemption for MAP

Physiological variables were obtained from the *apacheApsVar* table, which stores the value generating the highest APACHE points during the first 24 hours of ICU admission. For components whose APACHE point function is monotonic, this coincides with the clinically worst value. The point function for MAP is bidirectional, assigning points to both hypotensive and hypertensive deviations, so the stored value corresponds to the most deviant extreme in either direction. Its encoding is bidirectional by construction, and MAP was therefore left unconstrained.

##### Objective Function

To prevent weight collapse during optimization, a scale-anchoring penalty was introduced to maintain the SD of the raw score near a target value $\sigma_{0}$:


$$L(w)=LogLoss(y,p(w))+\alpha \sum_{j=1}^{d}\sum_{b=0}^{k_{j}-1}\frac{(w_{j,b}-{\hat{w}}_{j}{)}^{2}}{n_{j,b}+\epsilon }+\lambda_{scale}(SD(s)-\sigma_{0}{)}^{2}$$

where p(w) denotes the predicted probability from a logistic regression model fitted on the standardized score, $z_{i}\text{=}\left(s\left(x^{\left(i\right)}\right)\text{-}\bar{s}\right)\text{/}SD\left(s\right)$, $n_{j,b}$ is the number of observations in bin b of variable j, ${\hat{w}}_{j}\text{=}k_{j}^{\text{-}1}\sum_{b\text{=}0}^{k_{j}\text{-}1}w_{j,b}$, the small-interval penalty α discourages bins with limited empirical support. The sparse-bin term (second term) shrinks interval contributions toward the variable’s own mean in inverse proportion to the empirical support of each bin. Weights were centered within each variable before the penalty is applied because the objective is invariant to per-variable level shifts; penalizing the uncentered weights would penalize an arbitrary quantity and would do so unequally across constrained and unconstrained variables.

The scale penalty $\lambda_{scale}$ anchors the dispersion of the raw score s and breaks the near scale-invariance induced by score standardization. Consequently, the interval weights w were estimated by minimizing L(w).

##### Optimization

As limited-memory Broyden-Fletcher-Goldfarb-Shanno algorithm with bound constraints (L-BFGS-B) supports box constraints but not linear inequalities, the ordering constraints were imposed by reparametrization in terms of nonnegative increments. For $j\in J^{\text{+}}$,


$$w_{j,0}\text{=}0,w_{j,b}\text{=}\sum_{m\text{=}1}^{b}\delta_{j,m},\delta_{j,m}\geq 0$$

with the symmetric construction anchored at $w_{j,k_{j}\text{-}1}\text{=}0$ for $j\in J^{\text{-}}$. Fixing one bin per variable at 0 loses no generality, for the reason given earlier, and yields a weight table in which every organ system contributes zero in its most favorable category and positive points as it deteriorates, mirroring the presentation of the original SOFA score.

Integer weights were obtained through a 3-step procedure:

1. Continuous optimization under bounds: Data-driven initial weights were first projected onto the feasible monotonic set using isotonic regression. The resulting weights were expressed in terms of nonnegative increments $\delta_{j,m}$, which were subsequently optimized by minimizing the objective function L(w) using L-BFGS-B, with $\delta_{j,m}\geq 0$ enforced as lower-bound constraints.
2. Scale-matched rescaling and rounding: The optimized weights were rescaled so that the raw score achieved a target dispersion $SD(s)\approx \sigma_{0}$ and then rounded to the nearest integer. Rescaling by a positive constant and rounding are both nondecreasing maps, so monotonicity is preserved.
3. Discrete coordinate-wise local refinement: Finally, a coordinate descent–style local search was performed in the integer domain. Each integer coefficient was perturbed by ±1 (within -W,W), and the change was accepted if it improved the full objective and left the ordering constraints satisfied. Iterating this process yielded a locally improved integer solution, recovering part of the performance typically lost in naive rounding while keeping all coefficients strictly integral.

Class imbalance was handled through inverse prevalence weighting during weight optimization. Under directional constraints, the score is increasing in risk by construction, so the sign convention requiring a positive logistic calibration coefficient is satisfied automatically.

Hyperparameter selection was performed in 2 stages. In the first stage, the number of intervals $k_{j}$ for each variable was determined automatically using a penalized validation criterion. For each candidate $k\in \left[2,K_{max}\right]$, the penalized validation loss was computed as $LogLoss_{val}\left(k\right)\text{+}\gamma \cdot k$, where γ=0.000405 was calibrated so that adding 1 interval must reduce the validation log loss by at least this amount. Laplace smoothing with ε=0.01 was applied to bin-level mortality estimates to prevent zero-probability bins.

In the second stage, a grid search over the training and validation sets was conducted to select the following hyperparameters: minimum leaf size for discretization trees ∈{15,25,35,45}, which controls the minimum number of observations in any terminal node and prevents pathologically small bins; the integer weight bound W∈{10,20,100}, which constrains all weights to the range -W,W ensuring clinical plausibility; the logistic regression regularization parameter $\lambda_{\text{LR}}\in \{0.001,0.01,0.1,1,10,100,1000\}$, determining the inverse regularization strength $C\text{=}1\text{/}\lambda_{\text{LR}}$ in the calibration model; and the sparse-bin penalty α∈{0.01,0.1,0,1}.

Two hyperparameters were fixed based on preliminary experiments on the training set: the scale-anchoring penalty $\lambda_{\text{scale}}\text{=}5.0$, which prevents weight collapse during optimization; and the target standard deviation $\sigma_{0}\text{=}10$, which anchors the dispersion of the raw additive score and ensures that integer rounding produces meaningfully distinct scores across the patient population. The detailed architecture is presented in Figure 2.

**Figure 2.**
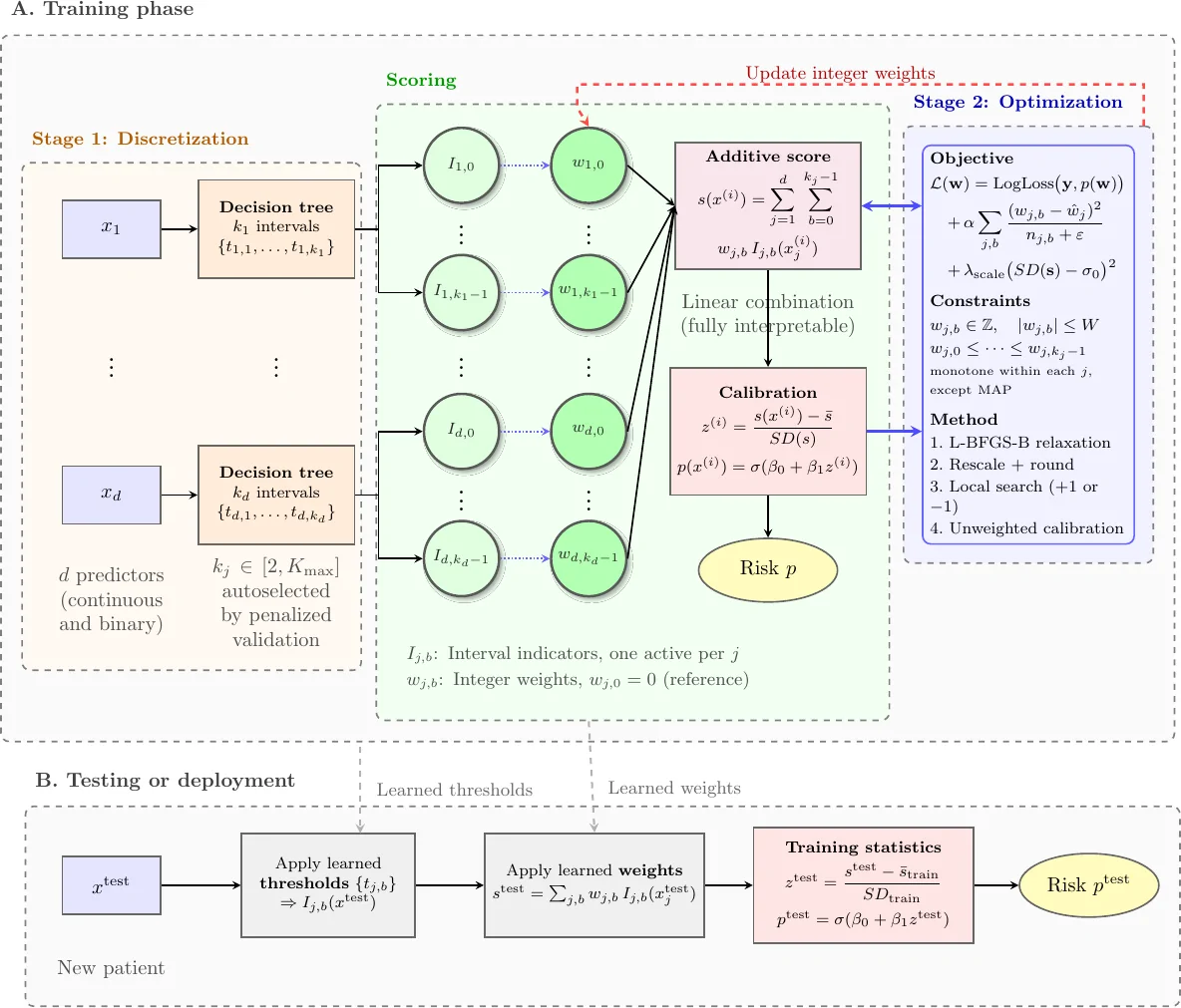
Overview of the proposed modeling framework. The training phase comprises feature discretization, additive integer-weight scoring, and optimization with calibration. In the testing or deployment phase, learned thresholds and weights are applied to new patients to compute the final risk estimate. MAP: mean arterial pressure.

### Statistical Analysis

Baseline characteristics were summarized overall and stratified by ICU survival status and by prespecified ICU length of stay (LOS) strata (2, 7, and 30 d). Continuous variables were reported as median with IQR; categorical variables were reported as counts and percentages. Between-group differences were assessed using the Mann-Whitney *U* test for continuous variables and the chi-square test for categorical variables. All tests were 2 sided, with *P* <.05 considered statistically significant.

Model performance was estimated using nested cross-validation grouped by hospital. Hospitals were assigned to 10 outer folds, with approximate stratification by ICU mortality and LOS distribution, ensuring that all encounters from a given hospital remained within the same fold and that no center contributed to both development and evaluation. Within each outer development set, hospitals were further partitioned into internal training and validation subsets, with approximately 25% of development encounters assigned to validation by whole hospitals. Candidate discretization schemes and model hyperparameters were fitted using the internal training data and selected according to validation log loss; the selected configuration was then refitted on the complete outer development set and evaluated once on the corresponding held-out hospitals. Each patient therefore contributed exactly one out-of-fold prediction, and pooled predictions were used to estimate overall performance. All comparator models were evaluated using the identical outer folds and equivalent hospital-grouped tuning procedures. Model selection and convergence criteria were prespecified before the revised analysis: configurations were selected by validation log loss, and runs with validation area under the receiver operating characteristic curve (AUROC) below 0.70 or with more than 3 predictors receiving identical weights across all their intervals were treated as nonconvergent. A single random seed was prespecified and held fixed throughout. Following completion of the hospital-grouped cross-validation, the final published score was refitted using the complete analytic cohort with the same prespecified development procedure. This full-cohort model was used only to derive the final thresholds and integer weights; it was not used to estimate predictive performance.

Discrimination was quantified using the AUROC and the area under the precision-recall curve (AUPRC), with 95% bias-corrected and accelerated bootstrap CIs from 5000 stratified resamples, computed on the pooled out-of-fold predictions. Paired comparisons between the recalibrated SOFA-derived score and the SOFA-derived reference score were performed on identical patients within each fold and summarized as the median and IQR of the paired difference across folds. Calibration was assessed using calibration curves comparing observed and predicted ICU mortality across risk strata, together with calibration slope, calibration-in-the-large, the observed-to-expected ratio (O:E), and the Brier score. The logistic calibration layer was fitted on unweighted development data within each fold, so that predicted probabilities correspond to the natural prevalence of the cohort. All analyses were performed in Python (version 3.10).

### Sensitivity Analysis for Missing Data

To assess the sensitivity of the findings to complete-case selection, we repeated the complete hospital-grouped nested cross-validation procedure after expanding the cohort to encounters missing no more than 1 of the 5 required continuous inputs. Under a rule specified before the sensitivity analysis was performed, a single missing bilirubin, creatinine, platelet, or MAP value was randomly assigned from a predefined range entirely contained within the corresponding zero-point category of the original SOFA: bilirubin, 0.2 to 1.1 mg/dL; creatinine, 0.4 to 1.1 mg/dL; platelets, 150 to 300×10⁹/L; and MAP, 70 to 95 mm Hg. Values were sampled from uniform distributions using a fixed random seed. This stochastic normal-range assignment avoided placing all recovered encounters at an identical arbitrary value while preserving the same prespecified assumption of no dysfunction in the affected SOFA domain. For nonventilated encounters with missing PaO_₂_/FiO_₂_, a ratio between 400 and 500 mm Hg was assigned under the same rule. Ventilated encounters with missing PaO_₂_/FiO_₂_ (n=5796) were not imputed because assuming normal oxygenation in the presence of mechanical ventilation was not considered clinically defensible. Encounters missing 2 or more required inputs were excluded. GCS was handled according to the same prespecified operational rule used in the primary analysis. The imputation procedure used no outcome information, and the complete model development and hospital-grouped validation procedure was then repeated in the expanded cohort.

### Ethical Considerations

Ethical approval from an institutional review board (IRB) or ethics committee was not required for this study. The eICU-CRD is a publicly available, deidentified critical care database that complies with the Health Insurance Portability and Accountability Act Safe Harbor provision for deidentification. Access to the database was granted through PhysioNet after the corresponding author completed the Collaborative Institutional Training Initiative program in human subjects research and signed a data use agreement. As this study involved secondary analysis of preexisting, fully anonymized data with no possibility of patient reidentification, it was deemed exempt from IRB review and individual informed consent requirements.

## Results

### Cohort Characteristics

A total of 14,848 ICU stays (153 hospitals) were included in the analysis, of which 2529 (17.03%) resulted in death and 12,319 (82.97%) in survival (Figure 1). Of 148,532 encounters with outcome information, 133,684 (90%) were excluded from the primary complete-case analysis because at least one required determination was unavailable. Missingness was concentrated in PaO_₂_/FiO_₂_ data and bilirubin, which were unavailable in 76.5% (113,595/148,532) and 64% (95,114/148,532) of encounters, respectively, compared with 19.6% (29,164/148,532) for creatinine, 8.8% (13,017/148,532) for platelet count, 1.54% (2290/148,532) for GCS, and 0.3% (481/148,532) for MAP. PaO_₂_/FiO_₂_ data and bilirubin were the sole missing determination in 27.4% (36,584/133,684) and 11.6% (15,477/133,684) of excluded encounters, respectively (Table S3 in Multimedia Appendix 1). Overall, 52,417 (39.2%) excluded encounters lacked exactly one required determination, whereas 81,267 (60.8%) lacked 2 or more. GCS was imputed according to the prespecified operational rule in 437 (2.9%) included encounters, of which 353 were mechanically ventilated. Urine output was available in 51.5% (76,447/148,532) of encounters with outcome information and was missing in 52% (7727/14,848) of the primary analytic cohort; requiring urine output would therefore have reduced the analytic cohort from 14,848 to 7121 encounters. The renal domain was consequently operationalized using creatinine.

Included and excluded encounters differed systematically in several measures of illness severity and treatment intensity (Table S3 in Multimedia Appendix 1). Mechanical ventilation was used in 64.2% (9531/14,848) of included compared with 20.2% (26,978/133,684) of excluded encounters (standardized mean difference [SMD] 0.995), norepinephrine in 28.6% (4243/14,848) versus 8.3% (11,153/133,684; SMD 0.540), and ICU mortality was 17% (2529/14,848) versus 4.5% (6066/133,684; SMD 0.411). Age was comparable between groups (absolute SMDs <0.03), and sex distributions were similar. Among included encounters, ICU mortality was 25.4% (111/437) among those with imputed GCS components and 16.8% (2,418/14,411) among those with recorded components.

Baseline characteristics, stratified by survival status and ICU LOS categories (≤2, ≤7, ≤30, and >30 d), are summarized in Table 2. Most deaths occurred early: 44.40% of nonsurvivors died within 2 days and 81.53% within 7 days. Among survivors, 27.94% were discharged within 2 days and 76.86% within 7 days, with only 118 (0.96%) remaining beyond 30 days.

**Table 2.** Baseline characteristics of the study cohort.

Variable	Nonsurvivor (n=2529, 17.03%)			Survivor (n=12,319, 82.97%)				*P* value		
	Length of stay ≤2 days (n=1123, 44.40%)	Length of stay ≤7 days (n=2062, 81.53%)	Length of stay ≤30 days (n=2529, 100%)	Length of stay ≤2 days (n=3442, 27.94%)	Length of stay ≤7 days (n=9469, 76.86%)	Length of stay ≤30 days (n=12,201, 99.04%)	Length of stay >30 days (n=118, 0.96%)	Length of stay ≤2 days	Length of stay ≤7 days	Length of stay ≤30 days
Age (y), median (IQR)	68 (56-78)	67 (56-77)	67 (56-77)	63 (51-74)	64 (53-74)	64 (52-74)	61 (50-71)	<.001	<.001	<.001
Sex, n (%)								.71	.04	.03
Male	613 (54.59)	1148 (55.67)	1424 (56.31)	1881 (54.65)	5137 (54.25)	6710 (55)	70 (59.32)			
Female	509 (45.33)	912 (44.23)	1103 (43.61)	1560 (45.32)	4331 (45.74)	5490 (45)	48 (40.68)			
Unknown	1 (0.09)	2 (0.10)	2 (0.08)	1 (0.03)	1 (0.01)	1 (0.01)	0 (0)			
PaO₂^a^ (mm Hg), median (IQR)	95 (66-166.50)	97.25 (69.90-159)	97 (70-156.10)	101 (78-147)	102 (77-151)	102 (76.80-152)	93 (72-145.80)	<.001	<.001	<.001
FiO₂^b^ (%), median (IQR)	100 (60-100)	90 (50-100)	80 (50-100)	40 (30-60)	50 (40-70)	50 (40-80)	80 (50-100)	<.001	<.001	<.001
Ventilation, n (%)								<.001	<.001	<.001
No	277 (24.67)	494 (23.96)	617 (24.40)	1772 (51.48)	3909 (41.28)	4670 (38.28)	30 (25.42)			
Yes	846 (75.33)	1568 (76.04)	1912 (75.60)	1670 (48.52)	5560 (58.72)	7531 (61.72)	88 (74.58)			
Respiratory rate (breaths/min), median (IQR)	35 (26-41)	34 (23-40)	33 (20-40)	29 (11-37)	29 (11-37)	30 (11-38)	30 (11-40)	<.001	<.001	<.001
GCS^c^, median (IQR)	6 (3-11)	6 (3-12)	7 (3-12)	14 (9-15)	12 (8-15)	11 (7-15)	9 (7-15)	<.001	<.001	<.001
GCS eyes, median (IQR)	1 (1-3)	1 (1-3)	2 (1-3)	4 (2-4)	3 (2-4)	3 (2-4)	3 (2-4)	<.001	<.001	<.001
GCS motor, median (IQR)	2 (1-6)	3 (1-6)	4 (1-6)	6 (5-6)	6 (4-6)	6 (4-6)	5 (4-6)	<.001	<.001	<.001
GCS verbal, median (IQR)	1 (1-3)	1 (1-3)	1 (1-4)	4 (1-5)	3 (1-5)	3 (1-5)	3 (1-5)	<.001	<.001	<.001
Mean arterial pressure (mm Hg), median (IQR)	47 (41-142)	52 (42-140)	53 (43-139)	63 (52-126)	62 (51-129)	61 (51-130)	58 (47.25-123)	<.001	<.001	<.001
Bilirubin (mg/dL), median (IQR)	1.10 (0.60-2)	0.92 (0.60-1.90)	0.90 (0.60-1.80)	0.60 (0.40-1)	0.60 (0.40-1.10)	0.70 (0.40-1.10)	0.85 (0.50-1.38)	<.001	<.001	<.001
Creatinine (mg/dL), median (IQR)	2.10 (1.40-3.10)	1.92 (1.20-2.93)	1.85 (1.17-2.88)	1.02 (0.72-1.61)	1.13 (0.75-1.85)	1.17 (0.75-1.93)	1.41 (0.89-2.13)	<.001	<.001	<.001
Platelets (×10⁹/L), median (IQR)	172 (100.50-242.50)	175 (107-245)	177 (109-246)	185 (139-245)	187 (137-247)	186 (135-249)	186 (142-252.75)	<.001	<.001	<.001
Heart rate (beats/min), median (IQR)	126 (105-143)	121 (103-139)	120 (102-138)	107 (92-122)	109 (94-125)	111 (96-128)	116.50 (98-139.75)	<.001	<.001	<.001
Dopamine, n (%)								<.001	<.001	<.001
No	973 (86.64)	1825 (88.51)	2257 (89.24)	3313 (96.25)	9061 (95.69)	11,655 (95.52)	108 (91.53)			
Yes	150 (13.36)	237 (11.49)	272 (10.76)	129 (3.75)	408 (4.31)	546 (4.48)	10 (8.47)			
Dobutamine, n (%)								<.001	<.001	<.001
No	1057 (94.12)	1939 (94.03)	2372 (93.79)	3397 (98.69)	9263 (97.82)	11,873 (97.31)	109 (92.37)			
Yes	66 (5.88)	123 (5.97)	157 (6.21)	45 (1.31)	206 (2.18)	328 (2.69)	9 (7.63)			
Epinephrine, n (%)								<.001	<.001	<.001
No	886 (78.90)	1702 (82.54)	2117 (83.71)	3354 (97.44)	9225 (97.42)	11,846 (97.09)	104 (88.14)			
Yes	237 (21.10)	360 (17.46)	412 (16.29)	88 (2.56)	244 (2.58)	355 (2.91)	14 (11.86)			
Norepinephrine, n (%)								<.001	<.001	<.001
No	480 (42.74)	913 (44.28)	1101 (43.53)	3027 (87.94)	7721 (81.54)	9441 (77.38)	63 (53.39)			
Yes	643 (57.26)	1149 (55.72)	1428 (56.47)	415 (12.06)	1748 (18.46)	2760 (22.62)	55 (46.61)			
SOFA^d^-derived reference score, median (IQR)	10 (8-13)	10 (7-12)	10 (7-12)	8 (6-10.75)	5 (4-7)	6 (4-8)	6 (4-8)	<.001	<.001	<.001

a PaO₂: partial pressure of arterial oxygen.

b FiO₂: fraction of inspired oxygen.

c GCS: Glasgow Coma Scale.

d SOFA: Sequential Organ Failure Assessment.

Across all LOS strata, nonsurvivors were consistently older than survivors (median age 67‐68 vs 61‐64 y; *P*<.001) and exhibited more severe organ dysfunction. Respiratory status was worse in nonsurvivors, with higher FiO_₂_ requirements and lower PaO_₂_ (all *P*<.001). GCS scores were markedly lower in nonsurvivors (*P*<.001). Hemodynamic and laboratory markers were also more severely abnormal: nonsurvivors had lower MAP, higher serum creatinine and bilirubin, lower platelet counts, and higher heart rate (all *P*<.001). Invasive mechanical ventilation and vasoactive support—particularly norepinephrine and epinephrine—were significantly more frequent among nonsurvivors (all *P*<.001). Sex distribution was similar between survivors and nonsurvivors in the ≤2-day stratum but differed in the ≤7- and ≤30-day strata (*P*=.04 and *P*=.03, respectively).

### Predictive Performance

#### Discrimination

Pooling the out-of-fold predictions across the 10 hospital-grouped folds, the recalibrated SOFA-derived score achieved an AUROC of 0.811 (95% CI 0.800‐0.819) compared with 0.751 (95% CI 0.740‐0.761) for the SOFA-derived reference score, corresponding to a paired improvement of 0.059 (95% CI 0.053‐0.067). AUPRC was 0.495 (95% CI 0.477‐0.515) for the recalibrated score and 0.393 (95% CI 0.377‐0.411) for the original score, with a paired improvement of 0.102 (95% CI 0.087‐0.116), against an observed event rate of 17.03%.

Across folds, AUROC ranged from 0.766 to 0.857 for the recalibrated score (median 0.807, IQR 0.791‐0.829) and from 0.713 to 0.791 for the original score (median 0.748, IQR 0.739‐0.772). Test folds contained between 494 and 3315 encounters drawn from 9 to 22 hospitals. The recalibrated score outperformed the original score in all 10 outer folds, with a median paired improvement of 0.057 AUROC (IQR 0.054‐0.062 range 0.036‐0.067) and 0.114 AUPRC (IQR 0.102‐0.124). Absolute AUROC varied by 0.091 across folds, whereas the paired difference varied by only 0.031, indicating that the relative improvement over the SOFA-derived reference score was substantially more stable than absolute discrimination across hospital groups.

Performance gains were also maintained across prespecified LOS strata (Figure 3). For ICU stays ≤2 days, AUROC was 0.924 (95% CI 0.914‐0.933) versus 0.866 (95% CI 0.852‐0.877) for the original score, with AUPRC 0.798 (95% CI 0.772‐0.820) versus 0.692 (95% CI 0.664‐0.716). For stays ≤7 days, AUROC was 0.855 (95% CI 0.845‐0.863) versus 0.793 (95% CI 0.781‐0.803), and AUPRC was 0.599 (95% CI 0.579‐0.619) versus 0.483 (95% CI 0.462‐0.503). For stays ≤30 days, AUROC was 0.812 (95% CI 0.802‐0.820) versus 0.752 (95% CI 0.741‐0.762), and AUPRC was 0.500 (95% CI 0.481‐0.519) versus 0.399 (95% CI 0.382‐0.418). The paired AUROC improvement was similar across these strata (+0.059, +0.062, and +0.059, respectively).

**Figure 3.**
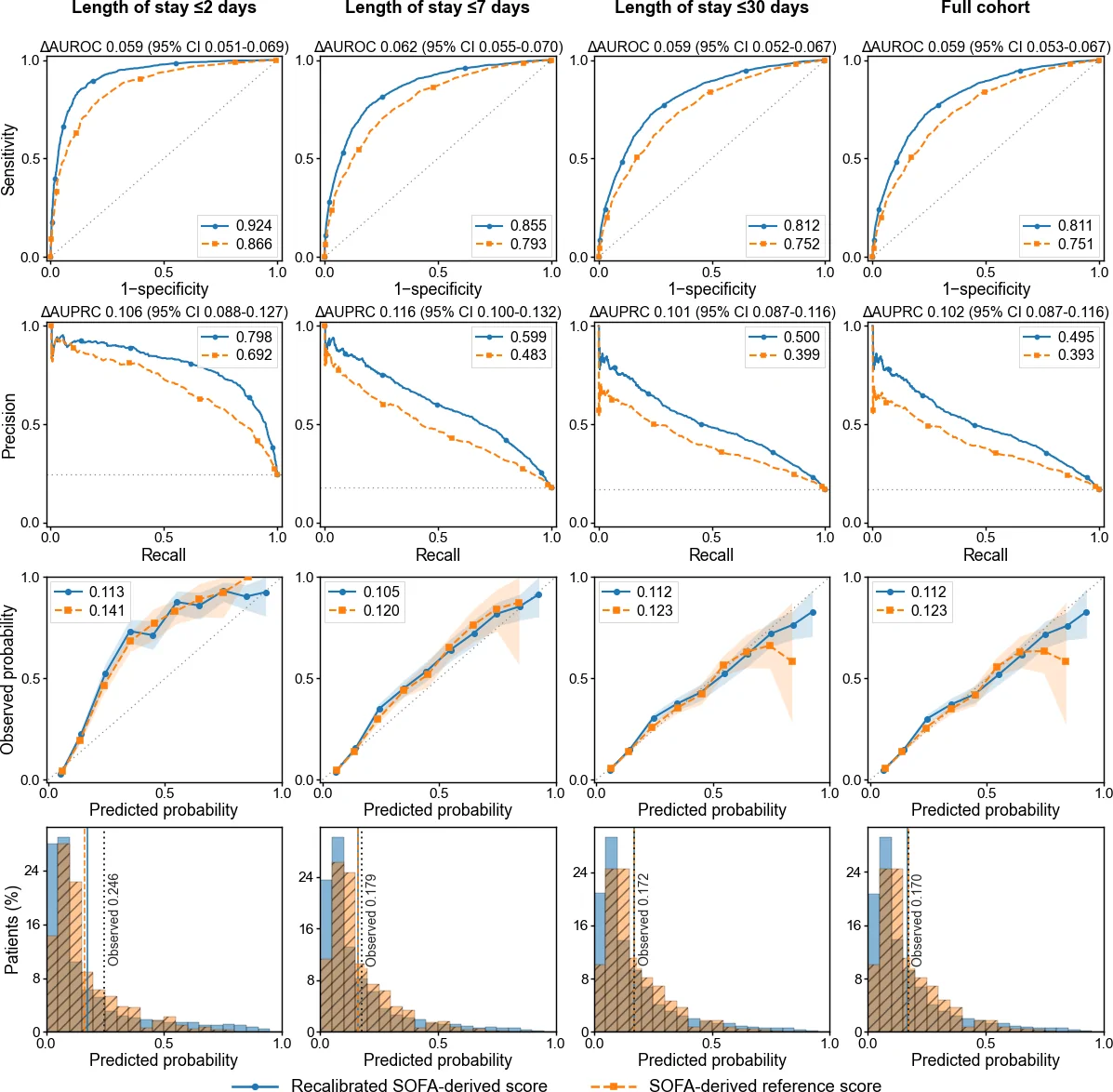
Discriminative performance, calibration, and risk stratification of the proposed SOFA-based model compared with the original SOFA-derived score, stratified by length of stay (LOS ≤2, ≤7, and ≤30 d) and in the overall cohort. The first row shows receiver operating characteristic (ROC) curves with the corresponding area under the curve and 95% CIs. The second row presents precision–recall curves with the AUPRC and 95% CIs. The third row displays calibration plots comparing predicted and observed mortality probabilities, with shaded areas indicating 95% CIs and Brier score for both models. The fourth row shows the distribution of predicted probabilities for each model, with vertical lines denoting the median predicted risk. Performance metrics are reported for each LOS stratum and for the overall cohort. AUPRC: area under the precision-recall curve; AUROC: area under the receiver operating characteristic curve; SOFA: Sequential Organ Failure Assessment.

Corresponding classification metrics across a range of probability thresholds are reported in Table S4 in Multimedia Appendix 1, with detailed subgroup-specific performance metrics provided in Table S5 in Multimedia Appendix 1.

#### Calibration

After unweighted logistic recalibration, the recalibrated score showed adequate global calibration on the pooled out-of-fold predictions (Figure 3). For the recalibrated score, the calibration slope was 1.011 (95% CI 0.970‐1.055), calibration-in-the-large was 0.008 (95% CI −0.042 to 0.053), and the observed-to-expected ratio was 1.005 (95% CI 0.973‐1.035), compared with 1.040 (0.990‐1.094), −0.006 (−0.053 to 0.036), and 0.996 (0.963‐1.026), respectively, for the original score. Mean predicted mortality closely matched the observed event rate of 17.03% for both models (16.9% for the recalibrated score and 17.1% for the original SOFA-derived score). The recalibrated score had a lower Brier score (0.112, 95% CI 0.109‐0.115) than the original score (0.123, 95% CI 0.119‐0.126). Global calibration remained adequate in the ≤30-day stratum, whereas larger deviations were observed in shorter-stay subgroups, particularly among patients with ICU stays ≤2 days, in whom both models underestimated observed mortality (O:E 1.400 for the recalibrated score and 1.507 for the original score). Additional calibration error measures and stratified estimates are provided in Table S6 in Multimedia Appendix 1. In the overall cohort, the recalibrated score had a lower Brier score but higher integrated calibration index, E50, E90, and E_max_ values than the SOFA-derived reference score. Taken together, these findings do not indicate consistently superior calibration relative to the reference score. Risk stratification by predefined mortality categories and associated outcomes are shown in Figure S1 in Multimedia Appendix 1.

#### Comparison With Alternative Models

To contextualize predictive performance, the proposed score was compared with penalized logistic regression, AutoScore, and XGBoost (Extreme Gradient Boosting) using the same candidate predictors and identical hospital-grouped outer folds, with model-specific tuning confined to the corresponding development data (Table 3). On pooled out-of-fold predictions, logistic regression achieved an AUROC of 0.775 and an AUPRC of 0.438, compared with 0.811 and 0.495, respectively, for the proposed score. AutoScore showed nearly identical discrimination to the proposed approach (AUROC 0.810), indicating that the principal distinction between the 2 interpretable methods lies in model structure rather than predictive performance: AutoScore derives a new additive score, whereas the proposed framework retains the predefined organ-domain structure of SOFA while updating its thresholds and integer weights. XGBoost achieved the highest discrimination, with an AUROC of 0.818 and an AUPRC of 0.510. Thus, the proposed additive score approached the performance of the more flexible nonlinear XGBoost model while maintaining an explicit point-based representation and direct attribution of each prediction to predefined clinical intervals.

**Table 3.** Comparison with alternative models.

Model^a^	Interpretable	AUROC^b^ pooled (95% CI)	AUPRC^c^ pooled (95% CI)	Δ AUROC vs SOFA^d^-derived, median (IQR)
SOFA-derived reference score	Yes	0.751 (0.740‐0.761)	0.393 (0.377‐0.411)	Reference
Logistic regression	Partial	0.775 (0.764‐0.784)	0.438 (0.419‐0.457)	0.029 (0.005‐0.042)
AutoScore	Yes	0.810 (0.799‐0.818)	0.490 (0.479‐0.509)	0.056 (0.054‐0.063)
Recalibrated SOFA-derived score	Yes	0.811 (0.800‐0.819)	0.495 (0.477‐0.515)	0.057 (0.054‐0.062)
XGBoost^e^	No	0.818 (0.807‐0.826)	0.510 (0.492‐0.529)	0.065 (0.063‐0.067)

a All models used the same candidate predictors and the same hospital-grouped outer folds.

b AUROC: area under the receiver operating characteristic curve.

c AUPRC: area under the precision-recall curve.

d SOFA: Sequential Organ Failure Assessment.

e XGBoost: Extreme Gradient Boosting.

### Final Model Structure and Learned Thresholds

After completion of the hospital-grouped cross-validation, the prespecified model development procedure was applied to the complete analytic cohort (n=14,848) to derive the final scoring instrument. The number of bins and model hyperparameters were selected using the prespecified internal validation procedure, after which the selected configuration was refitted on the full cohort. This final refit was used solely to define the thresholds and integer weights of the published score; all predictive performance estimates reported in this study were derived exclusively from out-of-fold predictions from the hospital-grouped cross-validation and not from the full-cohort fit.

The recalibrated SOFA-derived model learned data-driven discretization thresholds and interval-specific weights for all components. The number of intervals per variable ranged from 2 to 5, reflecting variable-specific granularity selected automatically by the algorithm. The model preserved a strictly additive and transparent structure in which each variable contributes through explicitly defined clinical ranges. Table 4 presents the complete threshold values and interval weights, along with those of the original SOFA-derived score. A direct comparison between the SOFA-derived reference and recalibrated discretization thresholds is provided in Figure S2 in Multimedia Appendix 1.

**Table 4.** SOFA-derived reference score and final recalibrated Sequential Organ Failure Assessment (SOFA)–derived score^a^.

Organ system and variable	SOFA-derived reference score, threshold (points)	Recalibrated score, threshold (weight)
Respiratory		
PaO₂^b^/FiO₂^c^ (mm Hg)	≥400 (0); <400 (1); <300 (2); <200+MV^d^ (3); and <100+MV (4)	>167.55 (0); 78.086‐167.55 (4); 49.9‐78.086 (10); and ≤49.9 (14)
MV	Included in respiratory criteria	Absent (0); present (3)
Neurological		
GCS^e^	15 (0); 13‐14 (1); 10‐12 (2); 6‐9 (3); and <6 (4)	>4.5 (0); and ≤4.5 (9)
Cardiovascular		
MAP^f^ (mm Hg)	>70 (0); ≤70+no vasoactive agent (1); ≤70+dopamine, dobutamine, or epinephrine without norepinephrine (2); ≤70+norepinephrine alone (3); and ≤70+norepinephrine plus any other vasoactive agent (4)	>138.5 (5); 51.5‐138.5 (0); 45.5‐51.5 (4); 40.5‐45.5 (5); and ≤40.5 (11)
Norepinephrine (binary)	Included in cardiovascular criteria	Absent (0); present (6)
Epinephrine (binary)	Included in cardiovascular criteria	Absent (0); present (6)
Dopamine (binary)	Included in cardiovascular criteria	Absent (0); present (2)
Dobutamine (binary)	Included in cardiovascular criteria	Absent (0); present (0)
Coagulation		
Platelets (×10⁹/L)	≥150 (0); <150 (1); <100 (2); <50 (3); and <20 (4)	>83.5 (0); 36.5‐83.5 (3); and ≤36.5 (7)
Renal		
Creatinine (mg/dL)	<1.2 (0); 1.2‐1.9 (1); 2.0‐3.4 (2); 3.5‐4.9 (3); and >5.0 (4)	≤1.085 (0); 1.085‐1.425 (2); 1.425‐1.665 (4); and >1.665 (6)
Hepatic		
Bilirubin (mg/dL)	<1.2 (0); 1.2‐1.9 (1); 2.0‐5.9 (2); 6.0‐11.9 (3); and >12.0 (4)	≤0.605 (0); 0.605‐1.245 (2); 1.245‐2.46 (3); 2.46‐8.05 (5); and >8.05 (7)

a Comparison of the prespecified reference score rules with the data-driven thresholds and integer weights learned by the proposed framework.

b PaO₂: partial pressure of arterial oxygen.

c FiO₂: fraction of inspired oxygen.

d MV: mechanical ventilation.

e GCS: Glasgow Coma Scale.

f MAP: mean arterial pressure.

The learned weights are not on the SOFA scale: the recalibrated SOFA-derived score ranges from 0 to 71 rather than 0 to 24, so its weights represent relative contributions within the fitted prognostic score rather than revised 0 to 4 organ-domain points. Maximum contributions were largest for the cardiovascular and respiratory predictor groups and smaller for the coagulation, hepatic, and renal domains. This partly reflects model structure because MAP, mechanical ventilation, and the 4 vasoactive agents enter as separate predictors rather than being combined within composite SOFA domains.

The direction of each contribution was imposed through the prespecified monotonicity constraints, whereas the cutpoint locations and relative magnitudes were estimated from the data. PaO_₂_/FiO_₂_, platelet count, and bilirubin showed ordered adverse-direction contributions, with the largest single interval contribution assigned to PaO_₂_/FiO_₂_ ≤49.9 mm Hg (Table 4). Creatinine contributions increased across thresholds of 1.085, 1.425, and 1.665 mg/dL. These outcome-derived cutpoints should not be interpreted as revised physiological definitions of respiratory, coagulation, hepatic, or renal dysfunction.

Other components departed more substantially from conventional SOFA grading. The neurological component retained a single cutpoint, assigning 9 points at GCS ≤4.5 and no additional points above this threshold. Thus, the fitted model concentrated neurological prognostic contribution at the most severe end of the GCS distribution rather than reproducing the graded neurological categories of SOFA. MAP remained bidirectional for the reason described in the *Methods* section: the APACHE-derived source field captures the first-day value associated with the greatest APACHE abnormality and may therefore represent either a hypotensive or hypertensive extreme. Among vasoactive therapies, norepinephrine and epinephrine each contributed 6 points, dopamine 2 points, and dobutamine 0 points. The zero dobutamine contribution should not be interpreted as evidence of clinical neutrality; it indicates that the fitted additive model assigned no additional prognostic contribution to this binary exposure variable. As vasoactive therapies were available only as binary indicators rather than harmonized dose measurements, these weights should be interpreted as cohort-specific prognostic contributions rather than dose-equivalent cardiovascular severity categories.

Overall, these cutpoints and weights characterize prognostic structure within this cohort under the prespecified model constraints and are not proposed as new physiological definitions of organ dysfunction. The recalibrated SOFA-derived score should therefore be interpreted as a mortality risk model rather than as a replacement for the descriptive organ dysfunction grading provided by conventional SOFA.

### Model Interpretability

The recalibrated SOFA-derived model retained full interpretability: each prediction decomposes into interval-specific contributions that sum to an additive score, which is then mapped to mortality risk through logistic calibration, ensuring a monotonic relationship between score and predicted mortality.

Several variables (PaO_₂_/FiO_₂_, GCS, and platelets) showed clear separation between extreme intervals, with adverse ranges receiving large positive contributions. Creatinine and bilirubin also displayed ordered increases in contribution across adverse ranges, whereas MAP showed a less ordered interval-specific profile. Vasoactive therapies, encoded as binary indicators, showed variable patterns: norepinephrine and epinephrine each contributed 6 points when present; dopamine contributed 2 points; and dobutamine contributed 0 points. Mechanical ventilation contributed 3 points when present.

The model remains fully transparent: changes in predicted risk can be attributed directly to specific clinical ranges and their additive contributions. From a clinical decision-making perspective, decision curve analysis was performed using the out-of-fold predictions from the hospital-grouped cross-validation and is shown stratified by LOS and overall cohort (Figure S3 in Multimedia Appendix 1).

### Predictive Stability

As a separate analysis of optimization stability, the entire model-development pipeline—automatic discretization, weight optimization, and integer projection—was repeated 1000 times using different random seeds for patient-level data partitioning and weight initialization. The original SOFA-derived score was evaluated on the identical test patients in each replication to enable paired comparison. All 1000 attempted replications satisfied the prespecified convergence criteria; none was rejected or repeated. Across replications, the recalibrated score achieved a median AUROC of 0.814 (IQR 0.808‐0.821; range 0.784‐0.839) and a median AUPRC of 0.502 (IQR 0.489‐0.514; range 0.438‐0.569), compared with 0.754 (IQR 0.747‐0.761; range 0.717‐0.780) and 0.387 (IQR 0.376‐0.397; range 0.341‐0.443), respectively, for the original score. The median paired improvement was 0.060 AUROC (IQR 0.055‐0.066; range 0.027‐0.088) and 0.116 AUPRC (IQR 0.106‐0.125 range 0.061‐0.153), with both differences positive in all 1000 replications. These replications characterize the stability of the optimization procedure under patient-level resampling and are distinct from the hospital-grouped cross-validation used for the primary performance assessment (Table 5).

**Table 5.** Predictive stability across 1000 patient-level resampling replications of the full optimization pipeline^a^.

Metric	Recalibrated SOFA^b^-derived model		SOFA-derived reference score		Paired improvement		Replications with improvement
	Median (IQR)	Range	Median (IQR)	Range	Median (IQR)	Range	
AUROC^c^	0.814 (0.808‐0.821)	0.784‐0.839	0.754 (0.747‐0.761)	0.717‐0.780	+0.060 (0.055‐0.066)	+0.027 to +0.088	1000/1000
AUPRC^d^	0.502 (0.489‐0.514)	0.438‐0.569	0.387 (0.376‐0.397)	0.341‐0.443	+0.116 (0.106‐0.125)	+0.061 to +0.153	1000/1000

a For each replication, the recalibrated and original SOFA-derived scores were evaluated on the identical test patients to enable paired comparison. All 1000 attempted replications satisfied the prespecified convergence criteria; none was rejected or repeated. This analysis characterizes optimization stability under resampling and is distinct from the hospital-grouped cross-validation used for the primary performance evaluation.

b SOFA: Sequential Organ Failure Assessment.

c AUROC: area under the receiver operating characteristic curve.

d AUPRC: area under the precision-recall curve.

### Structural Stability

The direction of the estimated interval weights was highly stable across the 1000 replications. Vasoactive drug weights also showed limited variability: norepinephrine had a median weight of 6 points (IQR 6‐6), epinephrine 5 (IQR 5‐6), dopamine 2 (IQR 2‐2), and dobutamine 0 (IQR 0‐1); mechanical ventilation contributed a median of 2 points (IQR 2‐2).

Cutpoint stability varied by predictor and ordinal position. Several boundaries were highly consistent, including the first GCS boundary (median 4.5, IQR 4.5‐4.5), the first MAP boundary (40.5 mm Hg, IQR 40.5‐40.5), and the first and fourth creatinine boundaries (1.09 mg/dL, IQR 1.09‐1.10; and 5.07 mg/dL, IQR 5.06‐5.13, respectively). Other boundaries, particularly for PaO_₂_/FiO_₂_, bilirubin, and platelets, showed greater variability or were selected in only a subset of replications.

Variation in individual cutpoints had a more limited effect on the resulting point functions. Across the evaluated predictor ranges, the mean interquartile width of the assigned points was 0.27 points for platelets, 0.55 for MAP, 0.65 for PaO_₂_/FiO_₂_, 0.71 for GCS, and 0.95 for creatinine. Bilirubin showed greater variability, with a mean interquartile width of 2.29 points.

At the patient level, total scores remained highly concordant across replications. The median within-patient IQR was 2 points on a median score range of 0 to 62, and 63.1% (9367/14,848) of patients had a score IQR of ≤2 points. The median pairwise Spearman correlation between scores generated by different replications was 0.970 (IQR 0.964‐0.975), indicating high stability of patient ranking despite variability in some individual cutpoints. Full distributions of cutpoints and weights are provided in Tables S7 and S8 in Multimedia Appendix 1.

### Sensitivity to the Complete-Case Requirement

The sensitivity analysis included 61,469 encounters from 186 hospitals, including 46,621 encounters with 1 imputed determination. We used the same 10-fold nested cross-validation procedure as in the primary analysis, with folds grouped by hospital. As the number of patients differed, the folds were redefined for this analysis. The recalibrated score achieved a higher AUROC than the SOFA-derived reference score in all 10 outer folds. Median AUROC was 0.853 (IQR 0.847‐0.857) for the recalibrated score and 0.818 (IQR 0.811‐0.821) for the original SOFA-derived score, corresponding to a median paired difference of 0.037 (IQR 0.034‐0.043; range 0.028‐0.046). Median AUPRC was 0.395 (IQR 0.372‐0.425) versus 0.300 (IQR 0.282‐0.320), with a median paired difference of 0.096 (IQR 0.085‐0.110). As the expanded cohort differed substantially from the primary complete-case cohort in case mix and event prevalence (8% vs 17%), this analysis was interpreted as a robustness assessment of the paired comparison rather than as a direct comparison of absolute performance between cohorts.

### Clinical Implementation Tool

To facilitate clinical adoption and prospective validation, we developed an open-source, web-based calculator that implements the recalibrated SOFA-derived score. The calculator is freely available online [25] and performs real-time computation of both the recalibrated score and mortality probability estimates. The interface accepts the same clinical variables used in the SOFA-derived reference score and applies the data-driven thresholds and weights derived in this study. All calculations are performed locally in the web browser, ensuring data privacy and enabling offline use. The calculator provides color-coded risk stratification and is designed for integration into existing clinical workflows, although this integration has not been evaluated prospectively. Source code for the calculator is included in the project repository to enable local deployment and adaptation to institution-specific requirements.

## Discussion

### Principal Findings

This study demonstrates that data-driven recalibration of a SOFA-derived score using optimization and ML components can substantially improve mortality prediction in contemporary ICU populations while preserving an additive, transparent, and interpretable structure. The recalibrated SOFA-derived score achieved consistent improvements in discrimination across all LOS strata, with AUROC gains of 6 percentage points and AUPRC improvements of 10 percentage points compared with the SOFA-derived reference score. Although global calibration was adequate after unweighted logistic recalibration, calibration was not consistently superior to that of the reference score. By learning data-adaptive thresholds and interval-specific contributions for each SOFA component, the proposed approach provides a mechanism for updating expert-defined scoring rules as clinical populations evolve [26,27] while preserving an additive structure grounded in SOFA-related clinical domains. As the recalibrated score uses its own 0 to 71 scale, treats several predictors separately, and does not reconstruct the original 0 to 24 SOFA score, it is not directly comparable with the canonical SOFA. The proposed framework provides an interpretable approach to updating SOFA-based risk scores.

Previous studies applying ML to ICU mortality prediction have reported AUROC values of 0.80 to 0.90, although often at the cost of interpretability [28]. Black box models face significant barriers to clinical adoption due to their opacity [18]. Interpretable approaches such as AutoScore [19] and FasterRisk [20] have demonstrated that transparent, point-based models can achieve competitive performance, but these methods construct entirely new scores rather than recalibrating existing instruments.

This work differs from de novo score construction in that it recalibrates a prespecified SOFA-derived structure rather than learning both the clinical framework and scoring architecture from scratch. Clinicians have decades of experience interpreting SOFA scores, and this familiarity represents institutional knowledge that would be lost with an entirely novel scoring system. The recent SOFA 2.0 [3] combined expert consensus with empirical analysis of more than one million ICU admissions, representing a major advance. However, its development required substantial expert involvement and resource-intensive consensus processes. Our automated, outcome-driven framework offers a complementary approach enabling more frequent updates as populations evolve.

From a methodological perspective, this work highlights the value of combining supervised ML with explicit structural constraints. Rather than replacing SOFA with a complex black box model, the proposed approach retains its familiar point-based logic and clinical interpretability [18], while allowing both thresholds and weights to be optimized from data [19]. The key innovation lies in the optimization of discretization boundaries and interval weights under constraints that preserve the additive, transparent structure of classical clinical scores. The integer constraint ensures manual computability; the scale-anchoring penalty prevents weight collapse; and automatic interval selection prevents overfitting while allowing variable-specific granularity. To our knowledge, this represents one of the first applications of a constrained optimization framework with ML components to recalibrate a prespecified SOFA-derived organ dysfunction score while preserving additivity and transparency.

Clinically, this recalibrated SOFA-derived model may offer a practical pathway for modernizing severity assessment in the ICU. As it relies on routinely collected SOFA-related variables and retains an additive structure, it could be integrated into existing clinical workflows or EHR systems with minimal friction.

The potential applications extend across multiple domains: (1) prognostication and family communication, where accurate risk estimates support shared decision-making [2]; (2) clinical trial enrollment and stratification, where improved discrimination enhances trial efficiency [29]; (3) quality benchmarking, where risk adjustment supports institutional comparisons [2]; and (4) sepsis identification, given SOFA’s central role in Sepsis-3 definitions [6].

Improved risk stratification achieved through transparent recalibration supports evidence-based decision-making without requiring clinicians to rely on opaque prediction models [18]. This alignment with the growing emphasis on explainable AI in medicine is crucial: regulatory bodies and frontline clinicians increasingly demand transparency in algorithmic decision support tools [18,30].

From an implementation perspective, the model’s computational requirements are minimal—only threshold evaluations and integer summations—enabling real-time deployment within existing EHR infrastructure without specialized hardware [19]. Unlike deep learning models requiring graphics processing unit acceleration, the recalibrated SOFA-derived score can be implemented as a simple lookup table, ensuring compatibility with legacy systems.

This additive structure may facilitate regulatory approval and clinical adoption under existing clinical decision support governance frameworks [18]. Future implementations should consider automated pipelines for periodic recalibration using local institutional data [27], enabling adaptive risk scores that maintain calibration as patient populations evolve [26]. The COVID-19 pandemic illustrated how rapidly populations can change; an automated recalibration framework could enable rapid adaptation to such shifts [31].

SOFA serves purposes beyond mortality prediction, including standardized description of organ dysfunction, sequential assessment, and its role in established clinical definitions such as Sepsis-3. This study addresses mortality risk estimation rather than these broader functions. Accordingly, the recalibrated SOFA-derived score is not intended to replace the canonical SOFA for organ dysfunction assessment. A potential future use would be to report the recalibrated risk score alongside conventional SOFA assessment when the required SOFA-related inputs are available electronically, without requiring a separate set of physiological measurements. This complementary use has not been evaluated in this study and would require external and prospective validation before clinical implementation.

It is important to distinguish periodic model updating from system-level adaptability. This study demonstrates the former: the score parameters can be reestimated using contemporary data while preserving the underlying additive framework. System-level adaptability, whereby the surrounding digital system can modify its behavior, content, or configuration in response to changing program requirements and user needs, represents a distinct property described in adaptable mobile health architectures [32] and was not evaluated in this study. Whether the recalibrated score can be effectively incorporated into clinical workflows, and whether the accompanying calculator supports real-world clinical use, requires prospective implementation and workflow evaluation.

Patterns of measurement availability can themselves carry predictive information in routinely collected ICU data [33,34]. We did not incorporate missingness indicators into the recalibrated score because the objective of this study was to update a prespecified SOFA-derived structure using physiological and treatment-related variables. Adding indicators of whether specific measurements were obtained would introduce a distinct measurement-process signal that may reflect local testing and documentation practices in addition to patient condition, potentially limiting transportability across hospitals [34,35]. Instead, we assessed the robustness of the findings to an alternative missing data assumption through the prespecified sensitivity analysis in the expanded cohort, in which the paired performance advantage was preserved. Explicitly modeling measurement processes together with physiology remains a distinct and potentially valuable direction for future prediction research.

Complete-case selection is an important limitation of this study. Encounters in which all required measurements were available differed from those in which 1 or more were unavailable. The high frequency of missing PaO_₂_/FiO_₂_ and bilirubin measurements, together with these between-group differences, is consistent with clinically selective measurement; however, the database does not establish why an individual measurement was or was not obtained. Complete-case selection may therefore have enriched the primary analytic cohort for more severely ill and intensively monitored patients and may affect both absolute and comparative performance estimates. Accordingly, the primary performance estimates should not be extrapolated directly to unselected ICU admissions. Nevertheless, the direction of the paired improvement over the SOFA-derived reference score was preserved in a prespecified sensitivity analysis that expanded the cohort to 61,469 encounters under an alternative normal-range imputation assumption for a single missing determination.

Real-world implementations of ML-based risk scores demonstrate that successful clinical integration depends on both technical performance and alignment with existing workflows [36]—tools perceived as disrupting established workflows face resistance regardless of predictive accuracy. By retaining an additive SOFA-derived structure, our approach may reduce this friction.

Future work will focus on external validation in independent cohorts, including international datasets such as MIMIC-IV, AmsterdamUMCdb, and HiRID, as well as extending the framework to longitudinal SOFA trajectories. Additional directions include periodic recalibration to address concept drift [26,27] and prospective evaluation of clinical utility in decision support and trial enrollment contexts. The framework will also be investigated for other additive scores, including APACHE, SAPS, the quick Sequential Organ Failure Assessment, National Early Warning Score, Model for End-Stage Liver Disease, and CURB-65 (confusion, urea, respiratory rate, blood pressure, and age ≥65 years).

This work should be interpreted in light of several limitations. First, the study is retrospective and based on a single database. Although eICU-CRD encompasses 208 US hospitals, external validation in independent international cohorts is required to confirm generalizability [24]. Second, the analysis focuses on static, day 1 SOFA assessment and does not capture temporal dynamics or trajectories of organ dysfunction. Trends in SOFA scores over time carry prognostic information beyond single-point assessments that our framework does not yet incorporate. Third, mortality was the sole outcome; impact on length of stay, functional outcomes, or treatment decisions remains unexplored. Fourth, we could not evaluate SOFA 2.0 as required variables were not consistently captured. Fifth, learned thresholds may reflect dataset-specific practice patterns [26], underscoring the need for validation in other settings. Sixth, because vasoactive-agent dose units could not be harmonized across hospitals and urine output was not incorporated, the reference comparator was a SOFA-derived operationalization rather than an exact reconstruction of the original SOFA. Its cardiovascular component used a prespecified ordinal mapping of MAP and binary vasoactive-agent administration, whereas its renal component used creatinine alone. Accordingly, performance comparisons in this study should be interpreted against this SOFA-derived reference score rather than the canonical original SOFA. Finally, this study evaluates discriminative performance but does not assess clinical utility prospectively.

### Conclusions

In summary, we have demonstrated that interpretable ML-based recalibration offers a practical and robust strategy for modernizing established clinical scores such as SOFA. By improving discrimination, achieving adequate global calibration after unweighted logistic recalibration, and maintaining transparency through an additive structure grounded in SOFA-related clinical domains, this approach provides a viable path toward responsible integration of ML into routine critical care assessment.

The framework demonstrates that the dichotomy between predictive performance and interpretability is false: with appropriate methodological design, meaningful improvements in risk prediction can be achieved without sacrificing transparency. This evolutionary approach—leveraging ML to optimize parameters of established instruments while preserving trusted structures—offers a promising route to realizing AI benefits in critical care while maintaining the accountability that patient safety demands.

## Supplementary material

10.2196/99361 Multimedia Appendix 1 Additional tables and figures.

## Abbreviations

APACHE: Acute Physiology and Chronic Health Evaluation
AUPRC: area under the precision-recall curve
AUROC: area under the receiver operating characteristic curve
CURB-65: confusion, urea, respiratory rate, blood pressure, and age ≥65 years
EHR: electronic health record
eICU-CRD: eICU Collaborative Research Database
FiO₂: fraction of inspired oxygen
GCS: Glasgow Coma Scale
ICU: intensive care unit
IRB: institutional review board
L-BFGS-B: limited-memory Broyden-Fletcher-Goldfarb-Shanno algorithm with bound constraints
LOS: length of stay
MAP: mean arterial pressure
MIMIC-IV: Medical Information Mart for Intensive Care IV
ML: machine learning
PaO₂: partial pressure of arterial oxygen
SAPS: Simplified Acute Physiology Score
SMD: standardized mean difference
SOFA: Sequential Organ Failure Assessment
XGBoost: Extreme Gradient Boosting
