# Supplementary material for: Interpretable Recalibration of a Sequential Organ Failure Assessment–Derived Score Using Machine Learning–Guided Optimization: Multicenter Cohort Study
Source: JMIR Med Inform. 2026 Nov 13;14:e99361. doi: 10.2196/99361 (PMC13766654; doi:10.2196/99361)
Supplement: Supplementary file 1 [file medinform-v14-e99361-s001.docx]

**Supplementary Material**

**Table S1. Sequential Organ Failure Assessment (SOFA) Score**

| **Organ System** | **Score** | | | | |
| --- | --- | --- | --- | --- | --- |
|  | **0** | **1** | **2** | **3** | **4** |
| **Respiration PaO₂/FiO₂, mmHg** | ≥400 | <400 | <300 | <200 with respiratory support | <100 with respiratory support |
| **Coagulation Platelets, ×10³/µL** | ≥150 | <150 | <100 | <50 | <20 |
| **Liver Bilirubin, mg/dL** | <1.2 | 1.2–1.9 | 2.0–5.9 | 6.0–11.9 | >12.0 |
| **Cardiovascular** | MAP ≥70 mmHg | MAP <70 mmHg | Dopamine <5 or dobutamine (any dose)* | Dopamine 5.1–15 or epinephrine ≤0.1 or norepinephrine ≤0.1* | Dopamine >15 or epinephrine >0.1 or norepinephrine >0.1* |
| **Central Nervous System Glasgow Coma Scale** | 15 | 13–14 | 10–12 | 6–9 | <6 |
| **Renal Creatinine, mg/dL or urine output** | <1.2 | 1.2–1.9 | 2.0–3.4 | 3.5–4.9 or <500 mL/d | >5.0 or <200 mL/d |

**Abbreviations:** FiO₂ = fraction of inspired oxygen; MAP = mean arterial pressure; PaO₂ = arterial oxygen partial pressure.

*Catecholamine doses are given as µg/kg/min for at least 1 hour.

Total SOFA score ranges from 0 to 24, with higher scores indicating more severe organ dysfunction.

**Reference:**

Singer M, Deutschman CS, Seymour CW, et al. The Third International Consensus Definitions for Sepsis and Septic Shock (Sepsis-3). *JAMA*. 2016;315(8):801-810. doi:10.1001/jama.2016.0287

**Table S2. Sequential Organ Failure Assessment (SOFA)-2 Score**

*Updated 2025*

| **Organ System** | **Score** | | | | |
| --- | --- | --- | --- | --- | --- |
|  | **0** | **1** | **2** | **3** | **4** |
| **Brain**  (GCS or motor  response) | GCS 15  (or thumbs-up,  fist, peace sign) | GCS 13-14  (or localizing to pain)  or drugs for delirium | GCS 9-12  (or withdrawal  to pain) | GCS 6-8  (or flexion  to pain) | GCS 3-5  (or extension to pain,  no response,  generalized myoclonus) |
| **Respiratory**  (PaO₂/FiO₂ or  SpO₂/FiO₂) | PaO₂/FiO₂ >300  mmHg (>40 kPa) | PaO₂/FiO₂ ≤300  mmHg (≤40 kPa) | PaO₂/FiO₂ ≤225  mmHg (≤30 kPa) | PaO₂/FiO₂ ≤150  mmHg (≤20 kPa)  AND advanced  ventilatory support* | PaO₂/FiO₂ ≤75  mmHg (≤10 kPa)  AND advanced  ventilatory support*  OR ECMO |
| **Cardiovascular**  (MAP and  vasopressors)† | MAP ≥70 mmHg  No vasopressor  or inotrope | MAP <70 mmHg  No vasopressor  or inotrope | Low-dose:  NE+Epi ≤0.2  µg/kg/min  OR any other  vasopressor/inotrope | Medium-dose:  NE+Epi >0.2-≤0.4  µg/kg/min  OR low-dose +  any other agent | High-dose:  NE+Epi >0.4  µg/kg/min  OR medium + other  OR mechanical  support‡ |
| **Liver**  (Bilirubin) | ≤1.2 mg/dL  (≤20.6 µmol/L) | ≤3.0 mg/dL  (≤51.3 µmol/L) | ≤6.0 mg/dL  (≤102.6 µmol/L) | ≤12.0 mg/dL  (≤205 µmol/L) | >12.0 mg/dL  (>205 µmol/L) |
| **Kidney**  (Creatinine or  urine output) | Creatinine  ≤1.2 mg/dL  (≤110 µmol/L) | Creatinine ≤2.0  mg/dL (≤170 µmol/L)  OR UO <0.5 mL/kg/h  for 6-12h | Creatinine ≤3.5  mg/dL (≤300 µmol/L)  OR UO <0.5 mL/kg/h  for ≥12h | Creatinine >3.5  mg/dL (>300 µmol/L)  OR UO <0.3 mL/kg/h  for ≥24h OR anuria  (0 mL) for ≥12h | Receiving or  meets criteria  for RRT  (includes chronic  dialysis)§ |
| **Hemostasis**  (Platelets) | >150 × 10³/µL | ≤150 × 10³/µL | ≤100 × 10³/µL | ≤80 × 10³/µL | ≤50 × 10³/µL |

**Abbreviations:** ECMO = extracorporeal membrane oxygenation; Epi = epinephrine; FiO₂ = fraction of inspired oxygen; GCS = Glasgow Coma Scale; MAP = mean arterial pressure; NE = norepinephrine; PaO₂ = arterial oxygen partial pressure; RRT = renal replacement therapy; SpO₂ = peripheral oxygen saturation; UO = urine output.

***Advanced ventilatory support:** High-flow nasal oxygen (HFNO), CPAP, BiPAP, non-invasive ventilation (NIV), invasive mechanical ventilation, or long-term home ventilation.

**†Vasopressor/inotrope scoring:** Only scored if administered as continuous IV infusion for at least 1 hour. Sum of norepinephrine and epinephrine doses (µg/kg/min). Other agents: dopamine, dobutamine, vasopressin, terlipressin, angiotensin II, phenylephrine, metaraminol.

**‡Mechanical support:** VA-ECMO, cardiac assist devices, intra-aortic balloon pump (IABP).

**§RRT criteria:** Includes patients currently receiving renal replacement therapy or meeting clinical criteria for initiation, including chronic dialysis.

**Note:** SpO₂/FiO₂ ratio can be used as alternative when PaO₂ is unavailable. Total score ranges from 0 to 24; higher scores indicate more severe organ dysfunction.

**Key Changes from SOFA-1 to SOFA-2:**

• Respiratory: SpO₂/FiO₂ ratio added as alternative; HFNO/NIV included in advanced support; ECMO added (score 4)

• Cardiovascular: Updated vasopressor thresholds; mechanical support devices included; expanded drug list

• Brain: Motor response alternatives; delirium treatment scores 1 point; myoclonus added to score 4

• Liver: Bilirubin thresholds adjusted (1-2 points modified)

• Kidney: Urine output criteria refined; RRT (including chronic) = 4 points

• Hemostasis: Platelet thresholds slightly adjusted (3-4 points)

**Reference:**

Ranzani OT, Singer M, Salluh JIF, et al. Development and Validation of the Sequential Organ Failure Assessment (SOFA)-2 Score. *JAMA*. 2025;334(23):2090-2103. doi:10.1001/jama.2025.20516

**Table S3. Missing-data accounting and comparison of encounters included in and excluded from the primary complete-case analysis**

a) **Availability and handling of score components among encounters with outcome information**

| **Score component** | **Missing or imputed, n (%)** | **Sole missing determination, n (% of exclusions)** | **Handling in primary analysis** |
| --- | --- | --- | --- |
| **PaO₂/FiO₂ data** | 113,595 (76.5%) | 36,584 (27.4%) | Excluded if unavailable |
| **Mean arterial pressure** | 481 (0.3%) | 5 (<0.1%) | Excluded if unavailable |
| **Bilirubin** | 95,114 (64.0%) | 15,477 (11.6%) | Excluded if unavailable |
| **Creatinine** | 29,164 (19.6%) | 97 (0.1%) | Excluded if unavailable |
| **Platelet count** | 13,017 (8.8%) | 254 (0.2%) | Excluded if unavailable |
| **Respiratory support** | 0 (0%) | 0 (0%) | - |
| **Vasopressors** | 0 (0%) | 0 (0%) | - |
| **Glasgow Coma Scale components** | 2,290 (1.5%) | — | Not an exclusion criterion; handled by prespecified operational rule |
| **GCS components imputed within included encounters** | 437 (2.9% of included) | — | 353 mechanically ventilated; 84 not mechanically ventilated |

Percentages in the second column are calculated among all 148,532 encounters with outcome information, except for the row 'GCS components imputed within included encounters', which uses the primary analytic cohort (n=14,848) as the denominator. Missingness categories overlap across variables and should not be summed.

b) **Clinical characteristics and treatment intensity of included and excluded encounters**

| **Variable** | **Included (n=14,848)** | **Excluded (n=133,684)** | **Standardized mean difference** |
| --- | --- | --- | --- |
| **ICU mortality** | 17.0% | 4.5% | 0.411 |
| **Mechanical ventilation** | 64.2% | 20.2% | 0.995 |
| **Norepinephrine** | 28.6% | 8.3% | 0.540 |
| **Epinephrine** | 5.3% | 1.2% | 0.230 |
| **Dopamine** | 5.6% | 1.8% | 0.200 |
| **Dobutamine** | 3.3% | 1.2% | 0.147 |
| **Age, years** | 64.0 [53.0–75.0] | 65.0 [53.0–76.0] | -0.025 |
| **Female sex** | 44.7% | 45.9% | — |
| **Heart rate** | 113.0 [96.0–130.0] | 104.0 [86.0–119.0] | 0.317 |
| **Respiratory rate** | 30.0 [12.0–38.0] | 27.0 [11.0–36.0] | 0.169 |
| **Temperature** | 36.4 [35.9–36.7] | 36.5 [36.2–36.7] | -0.223 |
| **Oxygen saturation, %** | 97.0 [94.0–99.0] | 97.0 [94.0–99.0] | -0.001 |

Notes: Continuous variables are shown as median [IQR] and categorical variables as percentages. Standardized mean differences are reported to describe between-group differences in case mix and treatment intensity.

**Table S4. Classification Metrics at Different Thresholds**

| **Probability Threshold** | **Model** | **Sensitivity** | **Specificity** | **PPV** | **NPV** | **Accuracy** | **F1-Score** | **LR+** | **LR-** | **Youden Index** | **TP** | **TN** | **FP** | **FN** |
| --- | --- | --- | --- | --- | --- | --- | --- | --- | --- | --- | --- | --- | --- | --- |
| **0,050** | **Original** | 0.983 | 0.118 | 0.186 | 0.972 | 0.265 | 0.313 | 1.11 | 0.14 | 0.101 | 2487 | 1448 | 10871 | 42 |
|  | **Recalibrated** | 0.969 | 0.244 | 0.208 | 0.974 | 0.367 | 0.343 | 1.28 | 0.13 | 0.213 | 2450 | 3003 | 9316 | 79 |
| **0.100** | **Original** | 0.888 | 0.392 | 0.231 | 0.944 | 0.476 | 0.366 | 1.46 | 0.29 | 0.280 | 2245 | 4829 | 7490 | 284 |
|  | **Recalibrated** | 0.857 | 0.573 | 0.292 | 0.951 | 0.622 | 0.436 | 2.01 | 0.25 | 0.431 | 2168 | 7062 | 5257 | 361 |
| **0.150** | **Original** | 0.726 | 0.654 | 0.301 | 0.921 | 0.666 | 0.426 | 2.10 | 0.42 | 0.380 | 1835 | 8059 | 4260 | 694 |
|  | **Recalibrated** | 0.762 | 0.720 | 0.358 | 0.936 | 0.727 | 0.487 | 2.72 | 0.33 | 0.482 | 1926 | 8872 | 3447 | 603 |
| **0.200** | **Original** | 0.601 | 0.762 | 0.341 | 0.903 | 0.734 | 0.435 | 2.52 | 0.52 | 0.363 | 1520 | 9385 | 2934 | 1009 |
|  | **Recalibrated** | 0.657 | 0.810 | 0.416 | 0.920 | 0.784 | 0.509 | 3.46 | 0.42 | 0.467 | 1662 | 9981 | 2338 | 867 |
| **0.250** | **Original** | 0.489 | 0.837 | 0.381 | 0.889 | 0.777 | 0.428 | 2.99 | 0.61 | 0.325 | 1236 | 10307 | 2012 | 1293 |
|  | **Recalibrated** | 0.549 | 0.869 | 0.462 | 0.904 | 0.814 | 0.502 | 4.19 | 0.52 | 0.418 | 1388 | 10705 | 1614 | 1141 |
| **0.300** | **Original** | 0.375 | 0.897 | 0.427 | 0.875 | 0.808 | 0.399 | 3.63 | 0.70 | 0.272 | 948 | 11046 | 1273 | 1581 |
|  | **Recalibrated** | 0.465 | 0.902 | 0.492 | 0.891 | 0.827 | 0.478 | 4.72 | 0.59 | 0.366 | 1175 | 11106 | 1213 | 1354 |
| **0.350** | **Original** | 0.293 | 0.937 | 0.487 | 0.866 | 0.827 | 0.366 | 4.63 | 0.76 | 0.229 | 740 | 11541 | 778 | 1789 |
|  | **Recalibrated** | 0.401 | 0.927 | 0.529 | 0.883 | 0.837 | 0.456 | 5.47 | 0.65 | 0.328 | 1015 | 11415 | 904 | 1514 |
| **0.400** | **Original** | 0.202 | 0.962 | 0.524 | 0.854 | 0.833 | 0.291 | 5.37 | 0.83 | 0.164 | 510 | 11856 | 463 | 2019 |
|  | **Recalibrated** | 0.344 | 0.944 | 0.555 | 0.875 | 0.841 | 0.425 | 6.08 | 0.70 | 0.287 | 869 | 11623 | 696 | 1660 |
| **0.450** | **Original** | 0.184 | 0.968 | 0.539 | 0.852 | 0.834 | 0.275 | 5.69 | 0.84 | 0.152 | 466 | 11920 | 399 | 2063 |
|  | **Recalibrated** | 0.293 | 0.958 | 0.586 | 0.868 | 0.844 | 0.391 | 6.90 | 0.74 | 0.251 | 741 | 11796 | 523 | 1788 |
| **0.500** | **Original** | 0.142 | 0.979 | 0.585 | 0.848 | 0.837 | 0.229 | 6.88 | 0.88 | 0.122 | 360 | 12064 | 255 | 2169 |
|  | **Recalibrated** | 0.252 | 0.969 | 0.628 | 0.863 | 0.847 | 0.360 | 8.22 | 0.77 | 0.222 | 638 | 11941 | 378 | 1891 |
| **0.550** | **Original** | 0.086 | 0.989 | 0.615 | 0.840 | 0.835 | 0.151 | 7.77 | 0.92 | 0.075 | 217 | 12183 | 136 | 2312 |
|  | **Recalibrated** | 0.214 | 0.976 | 0.651 | 0.858 | 0.847 | 0.322 | 9.07 | 0.81 | 0.190 | 540 | 12029 | 290 | 1989 |
| **0.600** | **Original** | 0.057 | 0.993 | 0.630 | 0.837 | 0.834 | 0.105 | 8.31 | 0.95 | 0.050 | 145 | 12234 | 85 | 2384 |
|  | **Recalibrated** | 0.174 | 0.984 | 0.694 | 0.853 | 0.846 | 0.278 | 11.05 | 0.84 | 0.158 | 440 | 12125 | 194 | 2089 |
| **0.650** | **Original** | 0.042 | 0.996 | 0.658 | 0.835 | 0.833 | 0.079 | 9.39 | 0.96 | 0.037 | 106 | 12264 | 55 | 2423 |
|  | **Recalibrated** | 0.140 | 0.989 | 0.723 | 0.849 | 0.844 | 0.235 | 12.71 | 0.87 | 0.129 | 355 | 12183 | 136 | 2174 |
| **0.700** | **Original** | 0.021 | 0.997 | 0.628 | 0.832 | 0.831 | 0.041 | 8.22 | 0.98 | 0.019 | 54 | 12287 | 32 | 2475 |
|  | **Recalibrated** | 0.112 | 0.992 | 0.745 | 0.845 | 0.842 | 0.195 | 14.26 | 0.89 | 0.104 | 284 | 12222 | 97 | 2245 |
| **0.750** | **Original** | 0.011 | 0.999 | 0.674 | 0.831 | 0.831 | 0.023 | 10.09 | 0.99 | 0.010 | 29 | 12305 | 14 | 2500 |
|  | **Recalibrated** | 0.084 | 0.994 | 0.749 | 0.841 | 0.839 | 0.151 | 14.54 | 0.92 | 0.078 | 212 | 12248 | 71 | 2317 |
| **0.800** | **Original** | 0.003 | 1.000 | 0.583 | 0.830 | 0.830 | 0.006 | 6.82 | 1.00 | 0.002 | 7 | 12314 | 5 | 2522 |
|  | **Recalibrated** | 0.057 | 0.997 | 0.775 | 0.837 | 0.837 | 0.107 | 16.82 | 0.95 | 0.054 | 145 | 12277 | 42 | 2384 |
| **0.850** | **Original** | 0.002 | 1.000 | 0.800 | 0.830 | 0.830 | 0.003 | 19.48 | 1.00 | 0.002 | 4 | 12318 | 1 | 2525 |
|  | **Recalibrated** | 0.033 | 0.999 | 0.838 | 0.834 | 0.834 | 0.063 | 25.27 | 0.97 | 0.032 | 83 | 12303 | 16 | 2446 |
| **0.900** | **Original** | 0.000 | 1.000 | 0.000 | 0.830 | 0.830 | 0.000 |  | 1.00 | 0.000 | 0 | 12319 | 0 | 2529 |
|  | **Recalibrated** | 0.013 | 0.999 | 0.829 | 0.831 | 0.831 | 0.026 | 23.66 | 0.99 | 0.013 | 34 | 12312 | 7 | 2495 |
| **0.950** | **Original** | 0.000 | 1.000 | 0.000 | 0.830 | 0.830 | 0.000 |  | 1.00 | 0.000 | 0 | 12319 | 0 | 2529 |
|  | **Recalibrated** | 0.002 | 1.000 | 1.000 | 0.830 | 0.830 | 0.005 |  | 1.00 | 0.002 | 6 | 12319 | 0 | 2523 |

**Abbreviations:** SOFA = Sequential Organ Failure Assessment; PPV = positive predictive value; NPV = negative predictive value; LR+ = positive likelihood ratio; LR− = negative likelihood ratio; TP = true positives; TN = true negatives; FP = false positives; FN = false negatives.

**Table S5. Detailed Performance Metrics Stratified by Subgroups**

| **Subgroup** | **AUROC** | | **AUPRC** | |
| --- | --- | --- | --- | --- |
|  | **Original** | **Recalibrated** | **Original** | **Recalibrated** |
| **Age** |  |  |  |  |
| **<50** | 0.788 [0.763–0.813] | 0.852 [0.830–0.872] | 0.356 [0.314–0.404] | 0.491 [0.442–0.541] |
| **50–64** | 0.753 [0.732–0.773] | 0.814 [0.797–0.832] | 0.381 [0.350–0.413] | 0.476 [0.444–0.514] |
| **65–74** | 0.739 [0.719–0.759] | 0.793 [0.774–0.811] | 0.408 [0.377–0.444] | 0.498 [0.462–0.534] |
| **>=75** | 0.740 [0.722–0.759] | 0.799 [0.783–0.817] | 0.448 [0.419–0.483] | 0.539 [0.510–0.573] |
| **Vasopressors** |  |  |  |  |
| **No** | 0.726 [0.710–0.742] | 0.783 [0.768–0.799] | 0.249 [0.229–0.271] | 0.336 [0.312–0.365] |
| **Yes** | 0.667 [0.650–0.685] | 0.748 [0.731–0.763] | 0.477 [0.457–0.501] | 0.583 [0.560–0.608] |
| **Mechanical Ventilation** |  |  |  |  |
| **No** | 0.765 [0.745–0.787] | 0.813 [0.795–0.831] | 0.325 [0.296–0.361] | 0.405 [0.372–0.443] |
| **Yes** | 0.734 [0.721–0.747] | 0.800 [0.789–0.811] | 0.417 [0.398–0.438] | 0.522 [0.502–0.545] |
| **Gender** |  |  |  |  |
| **Male** | 0.744 [0.729–0.760] | 0.805 [0.792–0.818] | 0.394 [0.372–0.419] | 0.492 [0.468–0.517] |
| **Female** | 0.761 [0.747–0.777] | 0.817 [0.804–0.831] | 0.397 [0.374–0.426] | 0.501 [0.474–0.530] |
| **Unknown** | 1.000 [1.000–1.000] | 1.000 [1.000–1.000] | 1.000 [1.000–1.000] | 1.000 [1.000–1.000] |
| **Sepsis** |  |  |  |  |
| **No** | 0.756 [0.743–0.769] | 0.822 [0.811–0.833] | 0.353 [0.332–0.375] | 0.467 [0.445–0.493] |
| **Yes** | 0.717 [0.698–0.735] | 0.770 [0.753–0.786] | 0.453 [0.425–0.481] | 0.536 [0.507–0.565] |

**Abbreviations:** SOFA = Sequential Organ Failure Assessment; AUCROC = area under the receiver operating characteristic curve; AUPRC = area under the precision–recall curve.

**Table S6. Calibration performance of the Original and Recalibrated SOFA-derived models overall and across length-of-stay strata**

| **Calibration performance by length-of-stay stratum** | | |
| --- | --- | --- |
| **Global** | | |
| **Metric** | **Recalibrated** | **Original** |
| **Calibration slope (95% CI)** | 1.011 (0.970–1.055) | 1.040 (0.990–1.094) |
| **CITL (95% CI)** | 0.008 (−0.042–0.053) | −0.006 (−0.053–0.036) |
| **O:E ratio (95% CI)** | 1.005 (0.973–1.035) | 0.996 (0.963–1.026) |
| **Brier score (95% CI)** | 0.112 (0.109–0.115) | 0.123 (0.119–0.126) |
| **ICI (95% CI)** | 0.016 (0.012–0.021) | 0.010 (0.006–0.015) |
| **E50 (95% CI)** | 0.012 (0.009–0.016) | 0.010 (0.005–0.015) |
| **E90 (95% CI)** | 0.032 (0.020–0.046) | 0.016 (0.009–0.028) |
| **Emax (95% CI)** | 0.072 (0.041–0.108) | 0.045 (0.015–0.094) |
| **Mean predicted risk (95% CI)** | 0.169 (0.166–0.172) | 0.171 (0.169–0.173) |
| **Observed event rate** | 0.170 | 0.170 |
| **LOS ≤2 days** | | |
| **Metric** | **Recalibrated** | **Original** |
| **Calibration slope (95% CI)** | 1.680 (1.575–1.796) | 1.762 (1.650–1.885) |
| **CITL (95% CI)** | 0.605 (0.535–0.671) | 0.613 (0.546–0.676) |
| **O:E ratio (95% CI)** | 1.400 (1.348–1.451) | 1.507 (1.446–1.567) |
| **Brier score (95% CI)** | 0.113 (0.106–0.119) | 0.141 (0.134–0.148) |
| **ICI (95% CI)** | 0.099 (0.090–0.107) | 0.100 (0.092–0.110) |
| **E50 (95% CI)** | 0.033 (0.028–0.038) | 0.028 (0.018–0.034) |
| **E90 (95% CI)** | 0.302 (0.276–0.329) | 0.300 (0.275–0.332) |
| **Emax (95% CI)** | 0.333 (0.308–0.364) | 0.326 (0.298–0.358) |
| **Mean predicted risk (95% CI)** | 0.176 (0.170–0.182) | 0.163 (0.159–0.167) |
| **Observed event rate** | 0.246 | 0.246 |
| **LOS ≤7 days** |  |  |
| **Metric** | **Recalibrated** | **Original** |
| **Calibration slope (95% CI)** | 1.250 (1.199–1.306) | 1.290 (1.225–1.358) |
| **CITL (95% CI)** | 0.156 (0.105–0.209) | 0.133 (0.084–0.183) |
| **O:E ratio (95% CI)** | 1.106 (1.070–1.143) | 1.101 (1.063–1.140) |
| **Brier score (95% CI)** | 0.105 (0.101–0.109) | 0.120 (0.116–0.124) |
| **ICI (95% CI)** | 0.035 (0.030–0.041) | 0.031 (0.026–0.037) |
| **E50 (95% CI)** | 0.020 (0.016–0.025) | 0.019 (0.014–0.024) |
| **E90 (95% CI)** | 0.094 (0.079–0.118) | 0.084 (0.067–0.102) |
| **Emax (95% CI)** | 0.107 (0.090–0.134) | 0.107 (0.080–0.142) |
| **Mean predicted risk (95% CI)** | 0.162 (0.158–0.165) | 0.162 (0.160–0.165) |
| **Observed event rate** | 0.179 | 0.179 |
|  |  |  |
| **LOS ≤30 days** | | |
| **Metric** | **Recalibrated SOFA** | **Original SOFA** |
| **Calibration slope (95% CI)** | 1.018 (0.977–1.060) | 1.049 (0.996–1.098) |
| **CITL (95% CI)** | 0.023 (−0.026–0.071) | 0.008 (−0.038–0.054) |
| **O:E ratio (95% CI)** | 1.015 (0.983–1.047) | 1.006 (0.973–1.039) |
| **Brier score (95% CI)** | 0.112 (0.109–0.116) | 0.123 (0.120–0.127) |
| **ICI (95% CI)** | 0.017 (0.012–0.022) | 0.010 (0.006–0.016) |
| **E50 (95% CI)** | 0.012 (0.009–0.016) | 0.011 (0.006–0.015) |
| **E90 (95% CI)** | 0.036 (0.023–0.051) | 0.017 (0.010–0.031) |
| **Emax (95% CI)** | 0.067 (0.038–0.102) | 0.037 (0.014–0.085) |
| **Mean predicted risk (95% CI)** | 0.169 (0.166–0.172) | 0.171 (0.169–0.173) |
| **Observed event rate** | 0.172 | 0.172 |

*Values are presented as point estimates with 95% confidence intervals (CI), unless otherwise indicated. Calibration slope values close to 1, calibration-in-the-large (CITL) values close to 0, and observed-to-expected (O:E) ratios close to 1 indicate better calibration. Lower Brier score, integrated calibration index (ICI), E50, E90, and Emax values indicate lower prediction error or calibration deviation. Mean predicted risk and observed event rate are expressed as proportions. LOS, length of stay; CI, confidence interval; CITL, calibration-in-the-large; O:E, observed-to-expected ratio; ICI, integrated calibration index; E50, median absolute calibration error; E90, 90th percentile absolute calibration error; Emax, maximum absolute calibration error.*

**Table S7. Stability of optimized cut-points, implied scoring functions, and patient-level scores across 1,000 resampling replications**

| **Predictor** | **Boundary position** | **Boundary selected, n/N (%)** | **Median** | **IQR** | **Range** |
| --- | --- | --- | --- | --- | --- |
| **PaO₂/FiO₂** | 1 | 1000/1000 (100.0%) | 53.52 | 49.90–67.01 | 47.39–107.07 |
| **PaO₂/FiO₂** | 2 | 999/1000 (99.9%) | 78.09 | 75.75–107.03 | 63.32–181.46 |
| **PaO₂/FiO₂** | 3 | 893/1000 (89.3%) | 123.55 | 107.15–167.55 | 78.09–185.05 |
| **PaO₂/FiO₂** | 4 | 549/1000 (54.9%) | 179.02 | 167.55–181.46 | 155.17–185.05 |
| **Glasgow Coma Scale** | 1 | 1000/1000 (100.0%) | 4.50 | 4.50–4.50 | 3.50–5.50 |
| **Glasgow Coma Scale** | 2 | 980/1000 (98.0%) | 7.50 | 7.50–8.50 | 4.50–8.50 |
| **Glasgow Coma Scale** | 3 | 437/1000 (43.7%) | 8.50 | 7.50–8.50 | 7.50–14.50 |
| **Glasgow Coma Scale** | 4 | 145/1000 (14.5%) | 9.50 | 8.50–14.50 | 8.50–14.50 |
| **Mean arterial pressure. mmHg** | 1 | 1000/1000 (100.0%) | 40.50 | 40.50–40.50 | 40.50–44.50 |
| **Mean arterial pressure, mmHg** | 2 | 1000/1000 (100.0%) | 45.50 | 42.50–45.50 | 41.50–51.50 |
| **Mean arterial pressure, mmHg** | 3 | 1000/1000 (100.0%) | 51.50 | 49.50–54.50 | 47.33–139.50 |
| **Mean arterial pressure, mmHg** | 4 | 887/1000 (88.7%) | 138.50 | 138.50–139.50 | 137.50–151.50 |
| **Platelets, ×10⁹/L** | 1 | 1000/1000 (100.0%) | 39.50 | 36.50–83.50 | 7.50–90.50 |
| **Platelets, ×10⁹/L** | 2 | 688/1000 (68.8%) | 83.50 | 83.50–83.50 | 34.50–90.50 |
| **Platelets, ×10⁹/L** | 3 | 151/1000 (15.1%) | 90.50 | 83.50–207.00 | 79.50–385.50 |
| **Creatinine, mg/dL** | 1 | 1000/1000 (100.0%) | 1.09 | 1.09–1.10 | 1.03–1.66 |
| **Creatinine, mg/dL** | 2 | 959/1000 (95.9%) | 1.42 | 1.42–1.66 | 1.33–5.13 |
| **Creatinine, mg/dL** | 3 | 894/1000 (89.4%) | 1.90 | 1.84–2.25 | 1.66–5.13 |
| **Creatinine, mg/dL** | 4 | 683/1000 (68.3%) | 5.07 | 5.06–5.13 | 4.84–5.14 |
| **Bilirubin, mg/dL** | 1 | 1000/1000 (100.0%) | 0.61 | 0.50–0.62 | 0.30–1.64 |
| **Bilirubin, mg/dL** | 2 | 947/1000 (94.7%) | 1.25 | 1.11–1.59 | 0.50–8.55 |
| **Bilirubin, mg/dL** | 3 | 739/1000 (73.9%) | 2.46 | 1.71–7.58 | 1.23–10.55 |
| **Bilirubin, mg/dL** | 4 | 425/1000 (42.5%) | 8.05 | 8.05–8.05 | 4.16–31.65 |

A) **Optimized cut-points by ordinal position**
B) **Stability of the implied scoring function**

| **Predictor** | **Median points range** | **Mean IQR width, points** | **Maximum IQR width, points** | **Grid with IQR ≤1 point, %** |
| --- | --- | --- | --- | --- |
| PaO₂/FiO₂ | 0–11 | 0,65 | 4.00 | 72.5 |
| Glasgow Coma Scale | 0–11 | 0.71 | 3.00 | 83.3 |
| Mean arterial pressure | 0–10 | 0.55 | 5.00 | 95.5 |
| Platelets | 0–6 | 0.27 | 4.00 | 93.8 |
| Creatinine | 0–7 | 0.95 | 2.00 | 95.5 |
| Bilirubin | 0–7 | 2.29 | 3.00 | 19.3 |

C) **Patient-level score concordance**

| **Stability measure** | **Result** |
| --- | --- |
| Median score range across replications | 0–62 points |
| Median within-patient IQR of total score | 2.0 points |
| Mean within-patient IQR of total score | 2.19 points |
| Patients with total-score IQR ≤1 point | 20.1% |
| Patients with total-score IQR ≤2 points | 63.1% |
| Median within-patient full range across replications | 18 points |
| Median pairwise Spearman correlation [IQR] | 0.970 [0.964–0.975] |

*Note: IQR = interquartile range; GCS = Glasgow Coma Scale. n/N denotes the number of replications in which a boundary at the indicated ordinal position was selected. Point-function stability was calculated from the replication-specific points assigned across the evaluated range of each continuous predictor. Patient-level score stability was assessed by applying the replication-specific scoring systems to the full analytic cohort.*

**Table S8. Stability of optimized interval weights across 1,000 replications**

| **Predictor** | **Interval/category** | **Replications, n** | **Weight, median [IQR]** | **Range** | **Directional consistency, %** |
| --- | --- | --- | --- | --- | --- |
| PaO₂/FiO₂ | Interval 1 | 1000 | 11 [10–12] | 6–16 | 100.0 |
| PaO₂/FiO₂ | Interval 2 | 1000 | 8 [6.00–9.25] | 0–12 | 100.0 |
| PaO₂/FiO₂ | Interval 3 | 999 | 4 [3–5] | 0–9 | 100.0 |
| PaO₂/FiO₂ | Interval 4 | 893 | 2 [0–3] | 0–5 | 100.0 |
| PaO₂/FiO₂ | Interval 5 | 549 | 0 [0–0] | 0–0 | 100.0 |
| Glasgow Coma Scale | Interval 1 | 1000 | 11 [10–11] | 9–12 | 100.0 |
| Glasgow Coma Scale | Interval 2 | 1000 | 4 [3–5] | 0–11 | 100.0 |
| Glasgow Coma Scale | Interval 3 | 980 | 0 [0–2] | 0–6 | 100.0 |
| Glasgow Coma Scale | Interval 4 | 437 | 0 [0–0] | 0–3 | 100.0 |
| Glasgow Coma Scale | Interval 5 | 145 | 0 [0–0] | 0–0 | 100.0 |
| Mean arterial pressure | Interval 1 | 1000 | 10 [10–11] | 7–13 | 100.0 |
| Mean arterial pressure | Interval 2 | 1000 | 6 [5–6] | 2–7 | 100.0 |
| Mean arterial pressure | Interval 3 | 1000 | 3 [2–4] | 0–5 | 100.0 |
| Mean arterial pressure | Interval 4 | 1000 | 0 [0–0] | 0–6 | 100.0 |
| Mean arterial pressure | Interval 5 | 887 | 4 [4–5] | 3–6 | 100.0 |
| Platelets | Interval 1 | 1000 | 6 [4–8] | 3–20 | 100.0 |
| Platelets | Interval 2 | 1000 | 3 [0–3] | 0–16 | 100.0 |
| Platelets | Interval 3 | 688 | 0 [0–0] | 0–13 | 100.0 |
| Platelets | Interval 4 | 151 | 0 [0–0] | 0–13 | 100.0 |
| Platelets | Interval 5 | 15 | 0 [0–0] | 0–0 | 100.0 |
| Creatinine | Interval 1 | 1000 | 0 [0–0] | 0–0 | 100.0 |
| Creatinine | Interval 2 | 1000 | 3 [2–3] | 1–6 | 100.0 |
| Creatinine | Interval 3 | 959 | 5 [4–6] | 2–7 | 100.0 |
| Creatinine | Interval 4 | 894 | 7 [6–7] | 5–9 | 100.0 |
| Creatinine | Interval 5 | 683 | 7 [6–7] | 5–9 | 100.0 |
| Bilirubin | Interval 1 | 1000 | 0 [0–0] | 0–0 | 100.0 |
| Bilirubin | Interval 2 | 1000 | 2 [2–2] | 1–5 | 100.0 |
| Bilirubin | Interval 3 | 947 | 4 [3–4] | 1–9 | 100.0 |
| Bilirubin | Interval 4 | 739 | 6 [5–7] | 3–10 | 100.0 |
| Bilirubin | Interval 5 | 425 | 7 [7–8] | 4–12 | 100.0 |
| Norepinephrine | Absent | 1000 | 0 [0–0] | 0–0 | 100.0 |
| Norepinephrine | Present | 1000 | 6 [6–6] | 4–7 | 100.0 |
| Epinephrine | Absent | 1000 | 0 [0–0] | 0–0 | 100.0 |
| Epinephrine | Present | 1000 | 5 [5–6] | 4–7 | 100.0 |
| Dopamine | Absent | 1000 | 0 [0–0] | 0–0 | 100.0 |
| Dopamine | Present | 1000 | 2 [2–2] | 1–3 | 100.0 |
| Dobutamine | Absent | 1000 | 0 [0–0] | 0–0 | 100.0 |
| Dobutamine | Present | 1000 | 0 [0–1] | 0–2 | 100.0 |
| Mechanical ventilation | Absent | 1000 | 0 [0–0] | 0–0 | 100.0 |
| Mechanical ventilation | Present | 1000 | 2 [2–2] | 1–4 | 100.0 |

*Weights are summarized across the stored patient-level resampling replications. For continuous predictors, interval numbering follows the ordinal bin index used by the optimization procedure. Note: IQR = interquartile range. Replications indicates the number of stored models in which the corresponding interval was present. Directional consistency is the proportion of those replications in which the optimized weight preserved the same direction according to the prespecified stability analysis.*

**Figure S1. Risk Stratification and Outcomes**

Risk stratification and calibration using predefined mortality risk categories: Low (0.00–0.20), Moderate (0.20–0.40), High (0.40–0.60), and Very High (0.60–1.01). Panel A shows patient distribution by risk group; Panel B compares predicted versus observed mortality with 95% confidence interval.

**Figure S2. Comparison of Thresholds - Original vs Recalibrated SOFA-derived score**

Distributions of SOFA components showing original SOFA intervals (red dashed) and recalibrated intervals with learned integer weights (blue solid). Grey histograms represent empirical variable distributions.

**Figure S3. Decision Curve Analysis**

Decision curve analysis comparing net benefit of original and recalibrated SOFA-derived score across probability thresholds, stratified by length of stay and overall cohort.
